# The δ subunit of F_1_F_o_-ATP synthase is required for pathogenicity of *Candida albicans*

**DOI:** 10.1038/s41467-021-26313-9

**Published:** 2021-10-15

**Authors:** Shuixiu Li, Yajing Zhao, Yishan Zhang, Yanli Zhang, Zhanpeng Zhang, Chuanyan Tang, Luobei Weng, Xiaohong Chen, Gehua Zhang, Hong Zhang

**Affiliations:** 1 Department of Dermatology, The First Affiliated Hospital of Jinan University, Guangzhou, Guangdong China; 2 Institute of Mycology, Jinan University, Guangzhou, Guangdong China; 3 Department of Otorhinolaryngology, The Third Affiliated Hospital of Sun Yat-Sen University, Guangzhou, Guangdong China

**Keywords:** Enzymes, Antifungal agents, Fungal pathogenesis, Pathogens

## Abstract

Fungal infections, especially candidiasis and aspergillosis, claim a high fatality rate. Fungal cell growth and function requires ATP, which is synthesized mainly through oxidative phosphorylation, with the key enzyme being F_1_F_o_-ATP synthase. Here, we show that deletion of the *Candida albicans* gene encoding the δ subunit of the F_1_F_o_-ATP synthase (*ATP16*) abrogates lethal infection in a mouse model of systemic candidiasis. The deletion does not substantially affect in vitro fungal growth or intracellular ATP concentrations, because the decrease in oxidative phosphorylation-derived ATP synthesis is compensated by enhanced glycolysis. However, the *ATP16*-deleted mutant displays decreased phosphofructokinase activity, leading to low fructose 1,6-bisphosphate levels, reduced activity of Ras1-dependent and -independent cAMP-PKA pathways, downregulation of virulence factors, and reduced pathogenicity. A structure-based virtual screening of small molecules leads to identification of a compound potentially targeting the δ subunit of fungal F_1_F_o_-ATP synthases. The compound induces in vitro phenotypes similar to those observed in the *ATP16*-deleted mutant, and protects mice from succumbing to invasive candidiasis. Our findings indicate that F_1_F_o_-ATP synthase δ subunit is required for *C. albicans* lethal infection and represents a potential therapeutic target.

## Abstract (web summary)

F_1_F_o_-ATP synthase is a key enzyme for energy production in fungi. Here, the authors show that the δ subunit of the enzyme is required for *Candida albicans* lethal infection and represents a potential therapeutic target.

## Introduction

Fungal infections, especially candidiasis and aspergillosis, claim an unacceptably high fatality rate, with an estimated 13 million people suffering from such life-threatening fungal infections worldwide and 2 million of them losing their lives annually^1^. Even worse, fungal infections account for nearly 50% of the 1.5 million AIDS-related deaths yearly^2^. Gaining insights into the mechanisms determining the lethal infections caused by this class of pathogens is therefore of considerable interest for uncovering novel treatment options.

*Candida* species are the second most prevalent agents that cause fungal infections worldwide, and *Candida albicans* is the most frequent pathogen isolated from patients suffering from severe fungal infections in the developed world^3^. Among the reported pathogenicity-related proteins, 60 are essential for *C. albicans* pathogenicity, and they are mainly involved in macromolecule synthesis, ion transport, signalling pathways, autophagy and energy metabolism (Supplementary Table 1). The ATP that is necessary for fungal cell growth and function is synthesized mainly through oxidative phosphorylation (OXPHOS), with the key enzyme being F_1_F_o_-ATP synthase^4^. However, it remains unclear how this enzyme affects pathogenicity. Notably, the structure of F_1_F_o_-ATP synthase from human pathogenic fungi has not been elucidated.

In *Saccharomyces*, the mitochondrial F_1_F_o_-ATP synthase produces the bulk of cellular ATP using the proton-motive force generated by respiratory chain complexes to pump protons across the inner membrane into the intermembrane space^5,6^. F_1_F_o_-ATP synthase consists of two major domains, a globular F_1_ catalytic domain (the α_3_β_3_ domain) and a membrane-bound F_o_ proton-translocating domain (the ab_2_c_8-12_ domain), which are linked by a central stalk containing the γ, ε and δ subunits^5,6^. The δ and ε subunits interact with a Rossmann fold in the γ subunit, forming a foot that interacts with the c-ring and couples the transmembrane proton motive force to catalyse in the α_3_β_3_ domain^7^, in which the δ subunit plays a key role in the mechanical coupling of the F_1_ domain to the F_o_ domain^6,8,9^. Based on the structural conservation of eukaryotic F_1_F_o_-ATP synthase, we homologously analysed *S. cerevisiae* F_1_F_o_-ATP synthase subunits in the UniProt and NCBI databases, and found that *C. albicans* F_1_F_o_-ATP synthase comprised at least 17 subunits (Supplementary Table 2), among which the δ subunit was encoded by the single exon *ATP16*.

Here, we report that the F_1_F_o_-ATP synthase δ subunit is required for lethal *C. albicans* infection. Deleting the δ subunit gene does not affect intracellular ATP levels, but reduces phosphofructokinase (Pfk1) activity, decreases fructose 1,6-bisphosphate (FBP) levels, blocks Ras1-dependent and -independent cAMP-PKA pathways, and downregulates virulence factors. Furthermore, a small molecule potentially targeting the δ subunit suppresses virulence factor expression in vitro and protects mice from morbidity and mortality upon systemic *C. albicans* infection.

## Results

### Deleting the δ subunit protects against lethal *C. albicans* infection

**Fig. 1:**
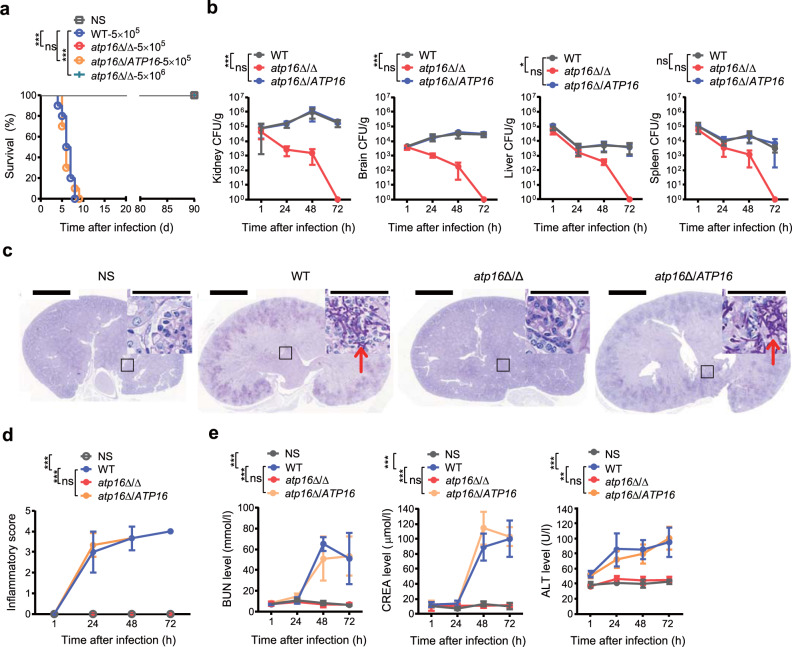
A mutant *C. albicans* strain carrying a deletion of the δ subunit fails to cause lethal infection. **a** Survival curves of mice (*n* = 10) after intravenous injection with NS and infection with *C. albicans* WT (5 × 10^5^ colony-forming units (CFU) per mice), *atp16*Δ/Δ (5 × 10^5^ CFU per mice), *atp16*Δ/*ATP16* (5 × 10^5^ CFU per mice), and *atp16*Δ/Δ (5 × 10^6^ CFU per mice). **b** *C. albicans* fungal burdens (CFU per g of tissue) in organs (*n* = 3) 1, 24, 48 and 72 h after intravenous infection with WT, *atp16*Δ/Δ, and *atp16*Δ/*ATP16* (5 × 10^5^ CFU per mice). **c**, **d** Representative images of periodic acid-Schiff- (PAS) stained kidney sections of mice (*n* = 3) 48 h after intravenous infection with WT, *atp16*Δ/Δ, and *atp16*Δ/*ATP16* (5 × 10^5^ CFU per mice) (**c**), and the combined inflammatory score based on renal immune cell infiltration (inflammation) and tissue destruction (*n* = 3) 1, 24, 48 and 72 h after intravenous infection with WT, *atp16*Δ/Δ, and *atp16*Δ/*ATP16* (5 × 10^5^ CFU per mice) (**d**). Arrowheads indicate fungal hyphae. Insets show higher-magnification images of the boxed areas; magnification ×10, ×400 (insets). Scale bars, 2000 µm, 50 µm (insets). **e** Blood urea nitrogen, creatine and alanine aminotransferase levels of mice (*n* = 3) 1, 24, 48 and 72 h after intravenous infection with WT, *atp16*Δ/Δ, and *atp16*Δ/*ATP16* (5 × 10^5^ CFU per mice). In **c** one representative experiment of three independent experiments is shown. In **a**, **b**, **d** and **e**, data are expressed as the mean ± SD. *P < 0.05, **P < 0.01, ***P < 0.001; ns, not significant; by log-rank test (**a**), or two-way ANOVA (**b**, **d**, **e**).

To test for the potential role of the δ subunit in lethal infection, we systemically challenged mice with a lethal dose^10,11^ of *C. albicans*. The survival rate of mice 90 days after infection with the δ subunit-encoding gene *ATP16* deletion mutant *atp16*Δ/Δ was 100%, while that of mice 8 days after infection with the wild-type strain WT was 0 (Fig. 1a). The survival rate of mice 90 days after infection with *atp16*Δ/Δ was still 100%, even if the challenged dose was increased to 10 times the above lethal dose (Fig. 1a). Likewise, etiologic analysis results showed that the fungal burdens in kidneys, brains, livers and spleens of the mice infected with *atp16*Δ/Δ gradually decreased and could not be detected at 72 h. The fungal burdens in livers and spleens of those mice infected with WT steadily decreased, whereas the fungal burdens in kidneys and brains continued to increase (Fig. 1b). Histopathologic analysis confirmed these results. The organs infected with *atp16*Δ/Δ showed normal morphology and no tissue damage, hyphae, pseudohyphae, spores or inflammatory cell infiltration and were not different from those of the normal saline (NS) control. The kidneys and brains of mice infected with WT displayed significant tissue damage with a mass of hyphae, the key virulence factor^12^, accompanied by a large number of inflammatory cells (Fig. 1c, d and Supplementary Fig. 1). Additionally, biochemical analysis showed that the BUN, CREA and ALT levels of mice infected with *atp16*Δ/Δ were not different from those of the NS control but were significantly lower than those of WT mice (Fig. 1e). These results indicate that δ subunit deletion abrogates *C. albicans*-mediated lethal infection.

**Fig. 2:**
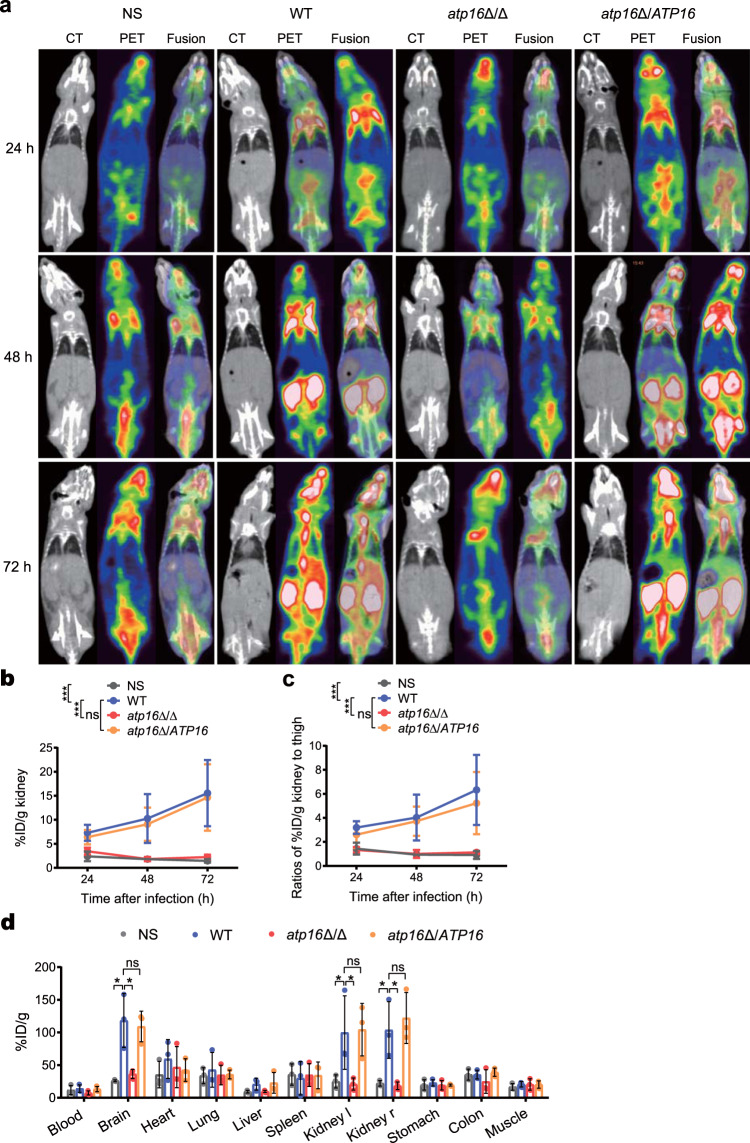
Deletion of the δ subunit abrogates lethal *C. albicans* infection as monitored by MicroPET. **a** CT, PET, and fused PET/CT images of NS-treated mice (*n* = 3) and WT-, *atp16*Δ/Δ-, and *atp16*Δ/*ATP16-*infected mice (*n* = 3) imaged with [^18^F]FDG (24, 48 and 72 h after infection). **b** Quantification of the in vivo PET signal intensity of NS-treated mice (*n* = 3) and WT-, *atp16*Δ/Δ-, and *atp16*Δ/*ATP16-*infected mice (*n* = 3) (24, 48 and 72 h after infection). **c** Normalization of the ratios of %ID/g in kidneys to those in the left thigh muscle (24, 48 and 72 h after infection). **d** The ex vivo biodistribution of [^18^F]FDG in the major organs of NS-treated mice (*n* = 3) and WT-, *atp16*Δ/Δ-, and *atp16*Δ/*ATP16-*infected mice (*n* = 3) injected with [^18^F]FDG (72 h after infection), as assessed with γ counter. Kidney l and Kidney r represent the left kidney and right kidney, respectively. In **a** one representative experiment of three independent experiments is shown. In **b**, **c** and **d**, data are expressed as the mean ± SD. *P < 0.05, ***P < 0.001; ns, not significant; by two-way ANOVA (**b**, **c**), or one-way ANOVA (**d**).

To further confirm the crucial role of the δ subunit in lethal infection, we constructed the complemented strain *atp16*Δ/*ATP16* and the overexpressed strain Atp16 O/E by reintroducing an *ATP16* gene ORF into the *ATP16* locus of *atp16*Δ/Δ and introducing an *ATP16* gene ORF into the *ADH1* locus of WT. Reintroduction of the δ subunit restored the ability of *atp16*Δ/Δ to cause lethal infection, as the mortality, fungal burdens, histopathology, damage scope and extent of mice infected with *atp16*Δ/*ATP16* were consistent with those of WT mice (Figs. 1 and 2 and Supplementary Fig. 1). However, δ subunit overexpression did not enhance lethal infection since the indices above in Atp16 O/E were not significantly different from those of WT (Supplementary Fig. 2). These results suggest that the δ subunit is required for lethal infection.

Furthermore, [^18^F]FDG-PET-imaging has been used to detect and monitor infection based on the significantly higher [^18^F]FDG uptake in active inflammatory cells than that in normal tissue cells^13,14^, so we used MicroPET/CT and γ counter to comprehensively monitor the dynamic changes of each organ, especially the targeted organ, the kidney, in mice after systemic infection. Consistent with the above results, the uptake of [^18^F]FDG in each tissue and organ of mice infected with *atp16*Δ/Δ was not different from that of the NS control but was significantly decreased compared to that of WT, especially in the kidneys (Fig. 2a). Then, we quantified the PET signal intensity biodistribution and found that the results were consistent with [^18^F]FDG-PET-imaging (Fig. 2b, c). To further estimate the scope of fungal damage, we assessed the ex vivo biodistribution of [^18^F]FDG in major organs with a γ counter and found that the results exhibited a similar trend (Fig. 2d). These results imply that δ subunit deletion results in the failure of *C. albicans* to activate inflammation in vivo.

### Deleting the δ subunit fails to cause major growth defects in *C. albicans*

Based on the observations of δ subunit pathogenicity in mice, we further explored the role of the δ subunit in fungal growth. To examine the in vitro growth, YPD medium with glucose as the glycolysis substrate and YPS medium with succinic acid as the OXPHOS substrate were used. In YPD medium, *atp16*Δ/Δ showed a similar lag phase, and the growth kinetics and the maximum growth rate achieved were slower and lower than those of WT (Supplementary Fig. 3a, b). In YPS medium, *atp16*Δ/Δ displayed remarkable growth defects compared to that of WT (Supplementary Fig. 3c, d). These results imply that the δ subunit is indeed a key protein of OXPHOS, and its deletion does not substantially affect in vitro growth of *C. albicans* if the glycolytic substrate is present.

To further examine the ex vivo growth, serum and organ homogenates were used. The growth of *atp16*Δ/Δ was weaker than that of WT, but the CFU value of *atp16*Δ/Δ at 24 h was 10- to 200-fold higher than its initial CFU value (Supplementary Fig. 3e). In the kidney and brain homogenates with 0.1% glucose, which is regarded as the physiological blood glucose concentration, the CFU value of *atp16*Δ/Δ at 24 h was 200-fold higher than the initial CFU value, and it did not have a significant difference from that of WT (Supplementary Fig. 3f, g). Consistently, to examine the in vivo growth, immunodeficient mice with suppressed macrophages were used, and the fungal burdens in kidneys and brains infected with *atp16*Δ/Δ continued to persist instead of decrease (Supplementary Fig. 3h, i). These results reveal that the δ subunit deletion allows *C. albicans* to become capable of growing and persisting in vivo but with reduced proliferation.

### Intracellular ATP levels are not affected by the δ subunit deletion

**Fig. 3:**
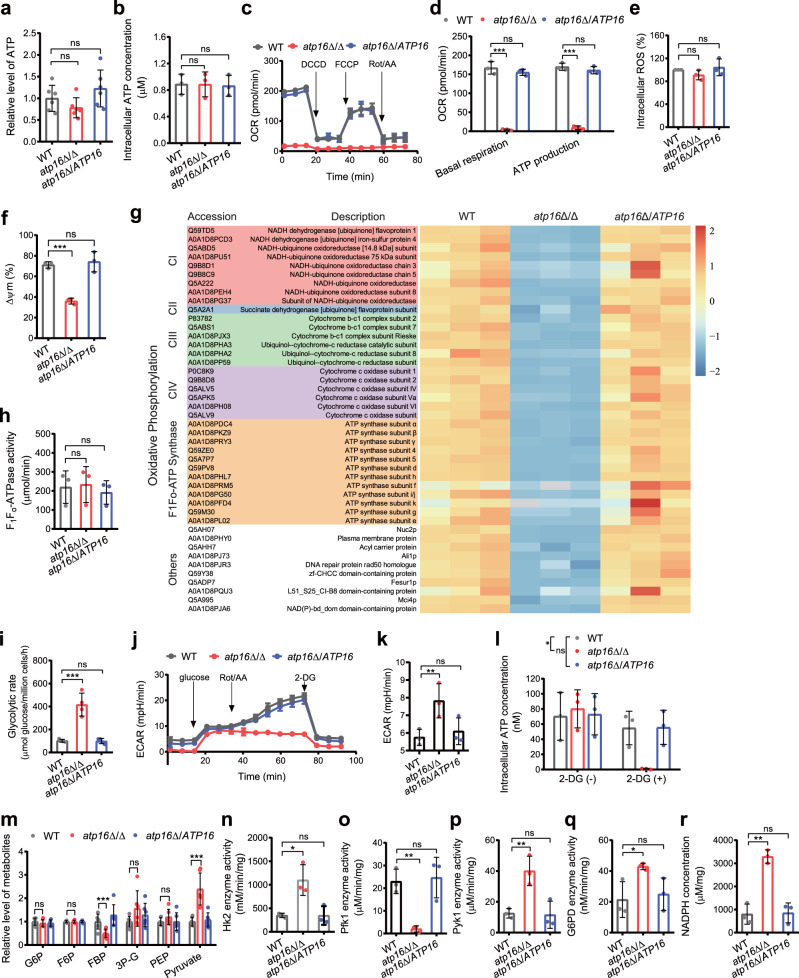
ATP synthesis in *C. albicans* lacking the δ subunit. **a** Intracellular ATP concentrations in WT, *atp16*Δ/Δ and *atp16*Δ/*ATP16* cultured in YNB + 2% glucose medium for 12 h were measured by using targeted metabolomics analysis. **b** The intracellular ATP content in WT, *atp16*Δ/Δ and *atp16*Δ/*ATP16* (2 × 10^6^ CFU) cultured in YPD medium for 12 h was measured by using luciferase/luciferin analysis. **c**, **d** Oxygen consumption rate (OCR) in WT, *atp16*Δ/Δ and *atp16*Δ/*ATP16* in response to dicyclohexylcarbodiimide (DCCD) (100 μM), Carbonyl cyanide 4-(trifluoromethoxy)phenylhydrazone (FCCP) (2 μM) and rotenone (Rot)/antimycin A (AA) (0.5 μM) were measured by a Seahorse XFe96 analyser (**c**) and bioenergetic parameters (basal respiration and OXPHOS ATP synthesis) were calculated (**d**). **e** ROS production in WT, *atp16*Δ/Δ and *atp16*Δ/*ATP16* were measured by a fluorometric assay. The ratios of the fluorescence intensities of the ROS-sensitive dyes were normalized to those in WT. **f** Mitochondrial membrane potential (ΔΨm) in WT, *atp16*Δ/Δ and *atp16*Δ/*ATP16* were measured by a fluorometric assay and the red/green mean fluorescent intensity ratio was calculated. **g** The expression levels of proteins involved in OXPHOS were assessed by proteomics analysis. The subunits of complex I, complex II, complex III, complex IV, F_1_F_o_-ATP synthase and others in *atp16*Δ/Δ were markedly downregulated compared with those in WT, fold change >1.5 (*p* < 0.05). The three columns for each strain represent three experiments were performed with 3 biological replicates. **h** F_1_F_o_-ATPase activity in WT, *atp16*Δ/Δ and *atp16*Δ/*ATP16*, were measured by an EnzChek Phosphate Assay Kit. **i** Glycolytic rate in WT, *atp16*Δ/Δ and *atp16*Δ/*ATP16*. **j**, **k**, Extracellular acidification rate (ECAR) in WT, *atp16*Δ/Δ and *atp16*Δ/*ATP16* in response to the glucose (10 mM), Rot/AA (0.5 μM) and 2-DG (50 mM) were measured by Seahorse XFe96 analyser (**j**) and bioenergetic parameters (glycolysis) were calculated (**k**). **l** Intracellular ATP content in WT, *atp16*Δ/Δ and *atp16*Δ/*ATP16* (2 × 10^5^ CFU) in the absence (−) or presence (+) of the glycolysis inhibitor 2-DG (50 mM) after cultured in YNB + 0.2% glucose medium at 30 °C for 12 h. **m** Glycolytic metabolites concentrations in WT, *atp16*Δ/Δ and *atp16*Δ/*ATP16* were measured by targeted metabolomics analysis. **n**, **o**, **p** The activities of the rate-limiting enzymes of glycolysis, Hk2 (**n**), Pfk1 (**o**), and Pyk1 (**p**), in WT, *atp16*Δ/Δ and *atp16*Δ/*ATP16*. **q**, **r** G6PD activity (**q**) and NADPH level (**r**) of the PPP pathway in WT, *atp16*Δ/Δ and *atp16*Δ/*ATP16*. In **a**, **m** six independent experiments were shown. In **b**–**f**, **h**–**l**, and **n**–**r** three independent experiments are shown. In **a**–**f** and **h**–**r** data were expressed as the mean ± SD. *P < 0.05, **P < 0.01, ***P < 0.001; ns, not significant; by two-tailed unpaired Student’s *t*-test (**a**, **b**, **d**–**i**, **k**, **m**–**r**), or two-way ANOVA (**l**).

To assess the effect of the δ subunit on intracellular ATP synthesis, we measured intracellular ATP content using targeted metabolomics (Fig. 3a) and luciferase/luciferin analysis (Fig. 3b), and found that there was no perceptible difference in the intracellular ATP content between the *atp16*Δ/Δ and WT. These results suggest that δ subunit is not required for sustaining intracellular ATP content.

To determine whether ATP synthesized through OXPHOS is affected by deletion of the δ subunit, we designed a scheme to measure the key points in mitochondrial oxidative respiration and the coupling process. The mitochondrial basal oxygen consumption rate (OCR) in *atp16*Δ/Δ was approximately 0 nmol O_2_/min/10^5^ cells, which was remarkably lower than that of WT (Fig. 3c, d). However, the amount of ROS produced in *atp16*Δ/Δ was similar to that of WT (Fig. 3e), indicating that δ subunit deletion causes *C. albicans* to stop oxidative respiration and the further production of extra ROS. The mitochondrial membrane potential (ΔΨm) in *atp16*Δ/Δ was markedly lower than that of WT (Fig. 3f), suggesting that δ subunit deletion leads to a weakened coupling function. The potential was calculated based on the mitochondrial oxygen consumption rate parameters, and it was found that ATP synthesis from OXPHOS in *atp16*Δ/Δ was ~6% of that in WT (Fig. 3d). These results indicate that δ subunit deletion impairs ATP synthesis from OXPHOS by abolishing oxidative respiration and coupling functions.

Furthermore, transmission electron microscopy (TEM) revealed that compared with WT, the mitochondrial inner membrane morphology in *atp16∆/∆* was partially disrupted, with a loss of partial cristae (Supplementary Fig. 4a). The quantitative reverse transcription PCR (RT-qPCR) results showed that mRNA levels of mitochondrial genes encoding OXPHOS subunits (including F_1_F_o_-ATP synthase proton channel core forming subunits a and c^9^) in *atp16∆/∆* were markedly reduced (Supplementary Fig. 4b), and the proteomics results showed that the expression levels of mitochondrial OXPHOS subunits (including F_1_F_o_-ATP synthase dimer formation contributing subunits i/j, e and g^15,16^) in *atp16∆/∆* were potently reduced (Fig. 3g). These results indicate that δ subunit deletion partially disrupts mitochondrial structure, downregulates mitochondrial OXPHOS DNA transcription and protein expression, and affects F_1_F_o_-ATP synthase proton channel assembly and dimer formation.

F_1_F_o_-ATP synthase not only synthesizes ATP but is also solely responsible for 80–90% of mitochondrial ATP hydrolytic activity^17^. The intracellular ATP content is determined by the rates of ATP synthesis and ATP hydrolysis^18^. Deletion of the δ subunit impairs ATP synthesis through OXPHOS, and whether δ subunit deletion reduces mitochondrial ATP hydrolysis remains unclear. To examine this, we measured the rate of mitochondrial ATP hydrolysis. Contrary to the expectation, the rate of mitochondrial ATP hydrolysis in *atp16*Δ/Δ displayed no significant difference from that of WT (Fig. 3h), suggesting that δ subunit deletion does not affect mitochondrial ATP hydrolysis.

It is known that a low ATP concentration is an activating signal for the rate-limiting enzymes of glycolytic flux^19^. To evaluate whether δ subunit deletion maintains intracellular ATP content by enhancing glycolysis, we evaluated alterations in the glycolysis process. First, the rate of glucose uptake (Fig. 3i) and extracellular acidification rate (ECAR) (Fig. 3j, k) in *atp16*Δ/Δ were 4-fold and 1.5-fold higher than those in WT, respectively, indicating that δ subunit deletion boosts glycolysis. Then, in the presence of the glycolysis inhibitor 2-deoxy-D-glucose (2-DG), the intracellular ATP content in *atp16*Δ/Δ lacking OXPHOS was almost 0, which was an approximately 84-fold decrease compared to that in the absence of 2-DG (Fig. 3l), suggesting that the intracellular ATP content in *atp16*Δ/Δ is mostly synthesized by glycolysis. Finally, targeted metabolomics analysis showed that *atp16*Δ/Δ enhanced the influx of glucose into glycolysis, which was characterized by both the depletion of glycolytic metabolites in the upstream of glyceraldehyde-3-phosphate dehydrogenase (GAPDH), especially FBP, and the hyperaccumulation of glycolytic metabolites in the downstream of GAPDH, especially pyruvate (Fig. 3m). These results imply that normal intracellular ATP levels are maintained in the δ subunit-deleted mutant due to enhanced glycolysis.

How does δ subunit deletion enhance glycolysis? RT-qPCR and proteomics results showed that mRNA (Supplementary Fig. 5a) and protein expression levels (Supplementary Fig. 5b) of glycolytic enzymes in *atp16*Δ/Δ were not significantly different from those in WT, indicating that δ subunit deletion does not affect the expression of glycolytic enzymes. Furthermore, we investigated the activities of the rate-limiting enzymes and found that the activities of Hk2 (Fig. 3n) and Pyk1 (Fig. 3p) in *atp16*Δ/Δ were 3-fold and 4-fold higher than those in WT respectively, while the activity of phosphofructokinase Pfk1 (Fig. 3o), the major regulatory enzyme that controls flux through glycolysis, was significantly reduced in *atp16*Δ/Δ. Nevertheless, the activity of G6PD (Fig. 3q), the key enzyme of the pentose phosphate pathway (PPP), in *atp16*Δ/Δ was much higher than that of WT, with an accumulation of the important metabolite NADPH (Fig. 3r). Together, these findings indicate that δ subunit deletion enhances glycolysis by increasing Hk2 and Pyk1 activity and compensating for the PPP pathway.

### The δ subunit is required for virulence of *C. albicans*

**Fig. 4:**
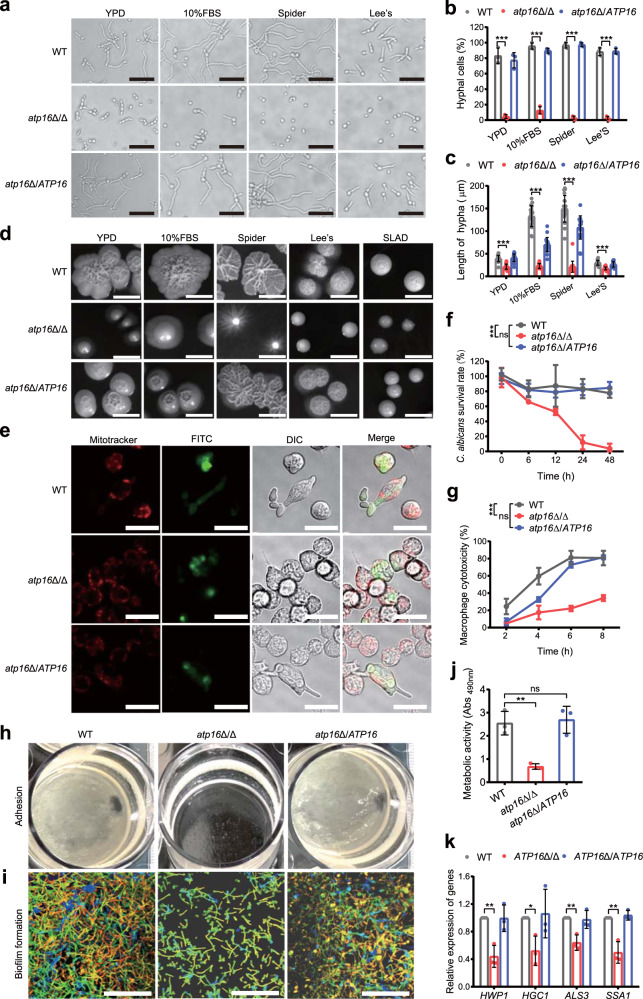
Deletion of the δ subunit causes defective virulence. **a**–**d** Hyphae formation of WT, *atp16*Δ/Δ and *atp16*Δ/*ATP16* were induced in YPD, 10% FBS, Spider, Lee’s liquid (2 h) (**a**) and solid (7 d) (**d**) media at 37 °C. In **a** magnification ×400. Scale bar is 50 µM. In **d** scale bar is 1 cm. The percentage of hyphal cells (**b**) and the length of hyphae (**c**) were calculated from at least 100 and 20 cells in each group, respectively. **e** RAW264.7 macrophages preloaded with MitoTracker Deep Red FM (red) were cocultured with FITC-stained WT, *atp16*Δ/Δ and *atp16*Δ/*ATP16* cells (green) at a 1:1 ratio in serum-supplemented DMEM and imaged after 3 h by confocal microscopy in the E_x644_/E_m655_ (red), E_x488_/E_m525_ (green) and DIC channels. Magnification ×630. Scale bar is 20 µM. **f** The *C. albicans* survival rates of WT, *atp16*Δ/Δ and *atp16*Δ/*ATP16* cells by the cocultured with RAW264.7 macrophages within 48 h were determined by the end point dilution assay at each time point. **g** Macrophage cytotoxicity caused by WT, *atp16*Δ/Δ and *atp16*Δ/*ATP16* were determined by the release of LDH. **h** Adhesion of WT, *atp16*Δ/Δ and *atp16*Δ/*ATP16* cells to the plastic plate bottom after 24 h of shaking incubation at 37 °C. **i**, **j** Biofilm formation of WT, *atp16*Δ/Δ and *atp16*Δ/*ATP16* after 6 h at 37 °C were observed by confocal microscopy in the E_x543_/E_m557_ channel (**i**), and their metabolic activity was determined by XTT assay (**j**). Magnification ×630. Scale bar is 20 µM. **k** The mRNA expression levels of the virulence-related genes of WT, *atp16*Δ/Δ and *atp16*Δ/*ATP16* cultured in Spider medium plus 0.2% glucose for 6 h as assessed by RT-qPCR. In **a**, **d**, **e**, **h** and **i** one representative experiment out of three independent experiments is shown. In **f**, **g**, **j** and **k** three independent experiments are shown. In **b**, **c**, **f**, **g**, **j** and **k** data expressed as the mean ± SD. *P < 0.05, **P < 0.01, ***P < 0.001; ns, not significant; by two-tailed unpaired Student’s *t*-test (**b**, **c**, **j**, **k**) and two-way ANOVA (**f**, **g**).

Among the known *C. albicans* virulence factors^20^, filamentation^21^ plays a key role in phagocytosis escape mediated through physical processes or by triggering NLRP3/caspase-1 inflammasome-dependent lysis^22,23^. As shown in the histopathologic analysis, filamentation could be observed in WT 1 h after infection but it was not observed in *atp16*Δ/Δ, indicating that the δ subunit is important for filamentation. To confirm this hypothesis, we performed a filamentation assay and found that in hyphal-inducing media, *atp16*Δ/Δ had severely impaired filamentation compared with WT (Fig. 4a–d). After phagocytosis by macrophages, *atp16*Δ/Δ was locked in only the spore form, with a significant reduction in the *C. albicans* survival rate and macrophage cell damage compared with that of WT, which formed hyphae and escaped from macrophages (Fig. 4e–g). As phagocytes are the first and central barrier against fungal systemic infection^24,25^, the filamentation defects of the δ subunit-deleted mutant likely facilitate its elimination by phagocytes.

We also measured other major virulence factors to comprehensively assess the contribution of the δ subunit to *C. albicans* virulence. Consistent with the filamentation defect, *atp16*Δ/Δ displayed defective adhesion (Fig. 4h), failed to form a three-dimensional biofilm (Fig. 4i) and showed obviously reduced metabolic activity (Fig. 4j). Moreover, *atp16*Δ/Δ displayed markedly downregulated hyphae-associated genes compared with that of WT (Fig. 4k). These results indicate that δ subunit deletion downregulates several virulence factors of *C. albicans*.

### δ subunit promotes the FBP-mediated cAMP-PKA pathway

**Fig. 5:**
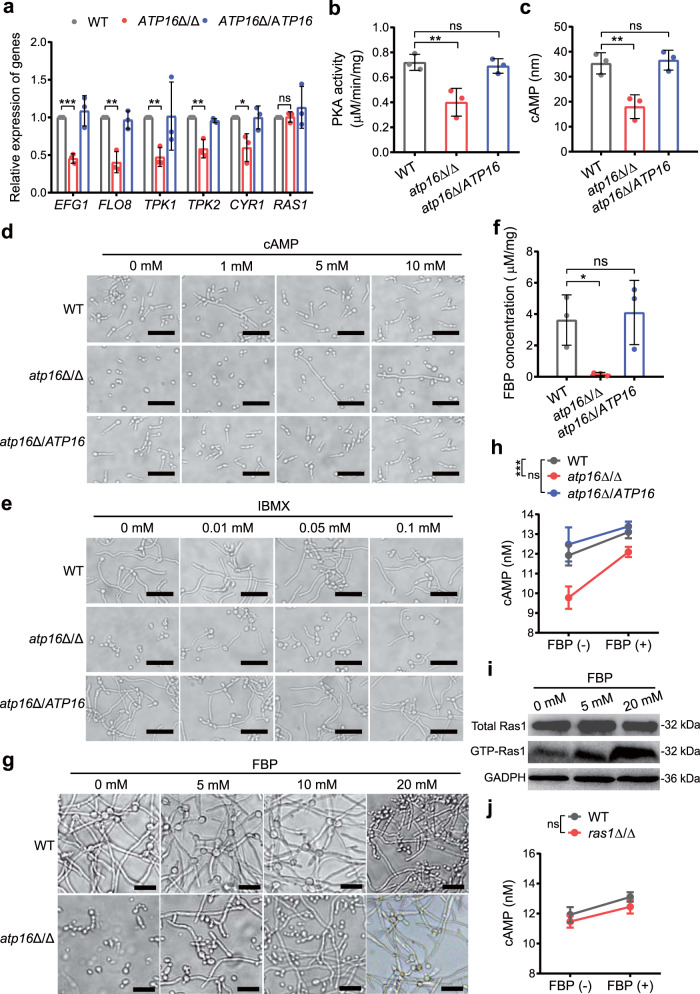
Deletion of the δ subunit blocks the FBP-mediated cAMP-PKA pathway. **a** Relative mRNA expression levels of the genes involved in the cAMP-PKA pathway in WT, *atp16*Δ/Δ and *atp16*Δ/*ATP16* were assessed by RT-qPCR. **b** PKA activities in WT, *atp16*Δ/Δ and *atp16*Δ/*ATP16* were assayed with colorimetric quantitative determination kits. **c** Intracellular cAMP concentrations in WT, *atp16*Δ/Δ and *atp16*Δ/*ATP16* (2 × 10^4^ CFU) were assayed with a cAMP-Glo™ Assay kit. **d**, **e** Hyphae formation of WT, *atp16*Δ/Δ and *atp16*Δ/*ATP16* in the presence of exogenous cAMP (0, 1, 5, 10 mM) (**d**) or 3-isobutyl-1-methylxanthine (IBMX) (0, 0.01, 0.05, 0.1 mM) (**e**) in Spider medium after incubation at 37 °C for 3.5 h. **f** Intracellular FBP concentration per mg protein in WT, *atp16*Δ/Δ and *atp16*Δ/*ATP16*. **g** Hyphae formation of WT and *atp16*Δ/Δ in the presence of exogenous FBP (0, 5, 10, 20 mM) in Spider medium after incubation at 37 °C for 3.5 h. **h** Intracellular cAMP concentrations of WT, *atp16*Δ/Δ and *atp16*Δ/*ATP16* (2 × 10^4^ CFU) in the absence (−) or presence (+) of FBP (20 μM). **i** GTP-Ras1 levels before and 3 min after the addition of different concentrations of FBP (0, 5, 20 mM) to permeabilized spheroplasts of *atp16*Δ/Δ. GAPDH was used as a loading control. **j** Intracellular cAMP concentrations of WT (SN250) and *ras1*Δ/Δ (2 × 10^4^ CFU) in the absence (−) or presence (+) of exogenous FBP (20 μM). Magnification ×400. Scale bar is 50 µM. In **a**, **b**, **c**, **f**, **h** and **j** data are expressed as the mean ± SD of three independent experiments. In **d**, **e**, **g** and **i** one representative experiment out of three independent experiments is shown. *P < 0.05, **P < 0.01, ***P < 0.001; ns, not significant; by two-tailed unpaired Student’s *t*-test (**a**, **b**, **c**, **f**), or two-way ANOVA (**h**, **j**).

It is known that the signalling pathways that regulate the virulence of *C. albicans* mainly include the cAMP-PKA pathway (including Ras1-dependent and Ras1-independent pathways), MAPK pathway, pH sensing pathway, negative regulators, matrix-embedded sensing pathway, and cell cycle arrest pathway^21,26^, among which the cAMP-PKA and MAPK pathways are the most important^27,28^. To clarify the underlying mechanism of the δ subunit in virulence, we first analysed the mRNA levels of the transcription factors in the key pathways. We found that the transcriptional levels of Cph1 and Tec1 in the MAPK pathway of *atp16*Δ/Δ were not significantly different from those of WT (Supplementary Fig. 6a), whereas the transcriptional levels of Efg1 and Flo8 in the cAMP-PKA pathway pronouncedly decreased (Fig. 5a). Therefore, we further detected alterations in the cAMP-PKA pathway. The RT-qPCR results showed that the upstream proteins Tpk1, Tpk2 and Cyr1 were consistently downregulated in *atp16*Δ/Δ (Fig. 5a). In addition, PKA phosphorylation, a necessary factor to regulate the downstream transcription factors Efg1 and Flo8, was reduced by 35% in *atp16*Δ/Δ with respect to that seen in WT (Fig. 5b). Furthermore, the intracellular cAMP content that induces PKA conformation shifts to stimulate its activity in *atp16*Δ/Δ was lower than that of WT (Fig. 5c). The addition of exogenous cAMP or treatment with 3-isobutyl-1-methylxanthine (IBMX), an endogenous activator of cAMP, led to concentration-dependent restoration of *atp16*Δ/Δ filamentation (Fig. 5d, e). Ras1, an upstream protein in this pathway, strongly reduced GTP-Ras1 activity in *atp16*Δ/Δ (81%) with respect to that of WT (Supplementary Fig. 6b). These results suggest that δ subunit deletion downregulates the virulence phenotype mainly via the cAMP-PKA pathway.

High or low intracellular ATP concentrations will activate or suppress the cAMP-PKA pathway, respectively^27,29^. However, δ subunit deletion does not affect the intracellular ATP concentration, indicating that δ subunit deletion suppresses the cAMP-PKA pathway by an alternative mechanism. The targeted metabolomics results showed that the glycolytic metabolites FBP and pyruvate in *atp16*Δ/Δ were most significantly shifted (Fig. 3m). Furthermore, the quantitative assay showed that intracellular FBP and pyruvate concentrations in *atp16*Δ/Δ were 22-fold lower (Fig. 5f) and 0.6-fold higher (Supplementary Fig. 7a) than those of WT, respectively. To specifically determine which molecule was working, the filamentation of permeabilized *C. albicans* cells was measured with exogenous FBP or pyruvate at physiological concentrations. FBP addition triggered the concentration-dependent enhancement of filamentation in *atp16*Δ/Δ (Fig. 5g), whereas the effect of pyruvate was minimal (Supplementary Fig. 7b), indicating that the δ subunit affects *C. albicans* virulence mainly via FBP. Therefore, we focused our study on FBP to ascertain its role in the cAMP-PKA pathway. As expected, the addition of exogenous FBP to *atp16*Δ/Δ triggered a rapid increase in the intracellular cAMP concentration (Fig. 5h), as well as the concentration-dependent enhancement of GTP-Ras1 activity (Fig. 5i). To test whether FBP regulates only the Ras1-dependent cAMP-PKA pathway, we repeated the above experiment on *ras1*Δ/Δ. We observed that exogenous FBP triggered an increase in intracellular cAMP concentration not only in *ras1*Δ/Δ but also in WT (Fig. 5j). These results imply that δ subunit deletion downregulates FBP-mediated Ras1-dependent and -independent cAMP-PKA pathways.

### δ subunit deletion interferes with Pfk1 activity by phosphorylation

**Fig. 6:**
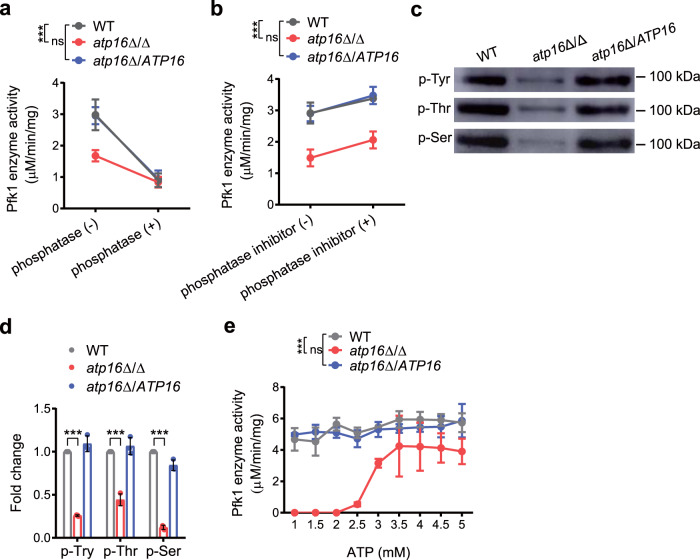
Deletion of the δ subunit attenuates Pfk1 activity via dephosphorylation to perturb its conformation. **a** Pfk1 activity of WT, *atp16*Δ/Δ and *atp16*Δ/*ATP16* in the absence (−) or presence (+) of alkaline phosphatase (2.5 U). **b** Pfk1 activity of WT, *atp16*Δ/Δ and *atp16*Δ/*ATP16* in the absence (−) or presence (+) of phosphatase inhibitor (5 μl). **c** Pfk1 phosphorylation levels were determined by immunoblotting after the enzymes purified by Affi-gel blue and ion-exchange chromatography from WT, *atp16*Δ/Δ and *atp16*Δ/*ATP16* with anti-phosphorylated tyrosine (p-Tyr) antibody, anti-phosphorylated threonine (p-Thr) antibody and anti-phosphorylated serine (p-Ser) antibody, respectively. **d** Quantification of Pfk1 p-Tyr, p-Thr and p-Ser phosphorylation in WT, *atp16*Δ/Δ and *atp16*Δ/*ATP16* by densitometry. **e** The activity of Pfk1 purified by polymer-bound Cibacron Blue F3G-A and eluted with different concentrations of ATP. In **a**, **b, d** and **e** data are shown as the mean ± SD of three independent experiments. In **c** one representative experiment out of three independent experiments is shown. ***P < 0.001; ns, not significant; by two-way ANOVA (**a**, **b** and **e**), or two-tailed unpaired Student’s *t*-test (**d**).

The intracellular FBP level and Pfk1 activity in *atp16*Δ/Δ were dramatically reduced to 4.5% and 9.1% of those measured in WT, respectively, and FBP is produced by the Pfk1-catalysed phosphorylation of fructose-6-phosphate, indicating that the δ subunit regulates the cAMP-PKA pathway through Pfk1-mediated FBP. We did not observe significant changes in the mRNA and protein expression levels of Pfk1 in *atp16*Δ/Δ compared to that of WT (Supplementary Fig. 5), indicating that δ subunit deletion does not affect Pfk1 transcription and translation. To assess whether the activity of Pfk1 was regulated by posttranslational modification, we enhanced or inhibited phosphorylation by adding either a phosphatase inhibitor or alkaline phosphatase to the tested strains and observed increased or decreased Pfk1 activity (Fig. 6a, b), indicating that phosphorylation regulates *C. albicans* Pfk1 activity. To further investigate whether the δ subunit affects Pfk1 phosphorylation, we purified Pfk1 and analysed its phosphorylation level between the tested strains by using anti-phosphoserine, anti-phosphothreonine and anti-phosphotyrosine antibodies. The serine/threonine and tyrosine phosphorylation of Pfk1 in *atp16*Δ/Δ were strongly decreased compared with that of WT (Fig. 6c, d), indicating that δ subunit deletion decreases Pfk1 phosphorylation.

Most intriguingly, during Pfk1 purification by affinity chromatography, we observed that the Pfk1 from WT bound to Cibacron Blue F3G-A could be eluted by low concentrations of ATP (0–2.5 mM); however, the Pfk1 from *atp16*Δ/Δ bound to Cibacron Blue F3G-A could not be eluted by ATP at the same concentrations (Fig. 6e), indicating that δ subunit deletion changes the Pfk1 conformation by enhancing its binding strength to Cibacron Blue F3G-A. Taken together, these findings indicate that δ subunit deletion reduces Pfk1 activity probably by inducing Pfk1 dephosphorylation, which causes conformation shifts.

### A compound potentially targeting the δ subunit inhibits *C. albicans* virulence in vitro and in vivo

**Fig. 7:**
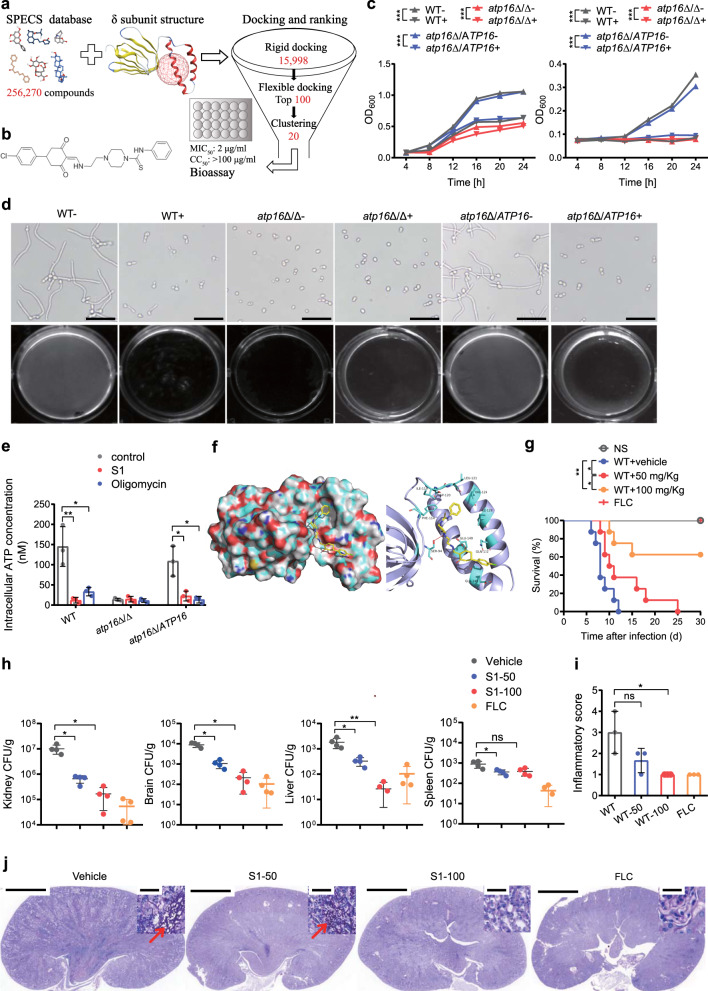
Identification of a compound potentially targeting the δ subunit that inhibits *C. albicans* pathogenicity. **a** Flowchart of the structure-based virtual screening strategy. A total of 256,270 compounds from the SPECS database were docked into the binding pocket (red grid) of the *C. albicans* δ subunit generated by homology modelling (PDB ID: 6CP3). After rigid and flexible docking and ranking, 20 compounds were selected to perform antifungal assays with WT, *atp16*Δ/Δ and *atp16*Δ/*ATP16* and cytotoxicity assays in liver (LO-2) and kidney (HK-2) cells. **b** The chemical structure of compound S1. **c** Growth of WT, *atp16*Δ/Δ and *atp16*Δ/*ATP16* in the absence (−) or presence (+) of S1 (10 μM) over time determined by cell density (OD_600_) in YPD (left) and YPS (right) media. **d** Hyphae formation of WT, *atp16*Δ/Δ and *atp16*Δ/*ATP16* in the absence (−) or presence (+) of S1 (10 μM) in Spider medium after 3 h of incubation at 37 °C. Magnification ×200; scale bars 100 µm. **e** Intracellular ATP content in WT, *atp16*Δ/Δ and *atp16*Δ/*ATP16* (2 × 10^6^ CFU) in the absence (control) or presence of S1 (10 µM) and oligomycin (10 µM) after cultured in YPS medium at 30 °C for 12 h. **f** The binding mode of the S1 and δ subunit. The N atom of S1 formed a hydrogen bond with the hydroxyl oxygen atom of the amino acid residue Ser94. **g** Survival curves of mice (*n* = 8) after intravenous infection with WT (2 × 10^5^ CFU per mice) without or with 7 days of S1 treatment (50 mg/kg and 100 mg/kg) and fluconazole (FLC) treatment (10 mg/kg) by oral gavage. **h** *C. albicans* fungal burdens (CFU per g tissue) in kidneys, brains, livers, spleens (*n* = 4) 8 days after intravenous infection with WT (2 × 10^5^ CFU per mice) with 7 days of S1 treatment (50 mg/kg and 100 mg/kg) and FLC treatment (10 mg/kg) by oral gavage. **i**, **j** Representative images of PAS-stained kidney sections of WT (2 × 10^5^ CFU per mice) infected mice (*n* = 3) with 7 days of S1 treatment (50 mg/kg and 100 mg/kg) and FLC treatment (10 mg/kg) by oral gavage (**j**), and the combined inflammatory score based on renal immune cell infiltration (inflammation) and tissue destruction (**i**). In **c**, **e** three independent experiments are shown. In **d**, **j** one representative experiment of three independent experiments is shown. In **c**, **e**, **g**, **h** and **i** data are shown as the mean ± SD. *P < 0.05, **P < 0.01, ***P < 0.001; by two-way ANOVA (**c**), log-rank test (**g**) or two-tailed unpaired Student’s *t*-test (**e**, **h** and **i**). In **j** Arrowheads indicate fungal hyphae. Insets show higher-magnification images of boxed areas; magnification ×10, ×400 (insets); scale bars, 2000 µm, 50 µm (insets).

Based on the above findings, it was reasoned that inhibiting the F_1_F_o_-ATP synthase δ subunit can terminate lethal *C. albicans* infection. Therefore, we set out to identify small molecules potentially inhibiting the δ subunit, by using structure-based virtual screening (SBVS). The receptor for SBVS was generated by homology modelling from the F_1_F_o_-ATP synthase δ subunit of *S. cerevisiae*. It displayed a β-sandwich-like architecture consisting of two β-sheets and two α-helices, on which there was a ligand binding pocket surrounded by the amino acid residues Pro76, Phe92, Ser94, Ala113, Phe114, Ile119, Asp120, Gln132 and Glu145 (Fig. 7a). After rigid and flexible molecular docking (using a database including >250,000 compounds), ranking and cluster analysis, 20 representative compounds were screened for in vitro antifungal activity verification. We prioritized the compound S1 (JNU-SM919, C_26_H_29_ClN_4_O_2_S) (Fig. 7b), a N-phenylpiperazine-1-carbothioamide for its significant inhibition of *C. albicans* growth (Fig. 7c).

As expected, 10 μM S1 only slightly inhibited the growth of WT in YPD medium but completely suppressed its growth in YPS medium (Fig. 7c). In addition, 10 μM S1 completely inhibited filamentation in Spider medium (Fig. 7d). The above phenotypes were similar to those of *atp16*Δ/Δ (Fig. 7c, d). S1 could not further weaken the above phenotypes of *atp16*Δ/Δ (Fig. 7c, d). Moreover, S1 inhibited the F_1_F_o_-ATP synthase activity of WT to a level close to that of *atp16*Δ/Δ, but did not affect the activity of *atp16*Δ/Δ (Fig. 7e). In silico analysis of the proposed interaction between S1 and the δ subunit suggests that the N atom of S1 might form a hydrogen bond with the Ser94 hydroxyl oxygen atom of the δ subunit (Fig. 7f). These results provide indirect evidence suggesting that S1 may act on the δ subunit.

The survival rate of mice treated with S1 30 days after systemic infection from WT was 60% (Fig. 7g), with the fungal burdens in kidneys, brains, livers and spleens significantly reduced 8 days after infection (Fig. 7h). Histopathologic analysis showed substantially reduced inflammation (Fig. 7i) and numbers of *C. albicans* hyphae, pseudohyphae and spores in the kidneys of S1-treated mice compared to those of untreated controls (Fig. 7j). Thus, S1 can inhibit lethal *C. albicans* infection.

## Discussion

Here, we showed that the F_1_F_o_-ATP synthase δ subunit is required for lethal infection in *Candida albicans*. Deletion of the δ subunit gene downregulated major virulence factors in vitro and abrogated lethal *C. albicans* infection in mice, without causing remarkable defects in intracellular ATP concentrations or in vitro growth. Furthermore, we identified a compound potentially targeting the δ subunit that induces similar effects.

The growth ability^30–32^ and virulence factor production^31^ of *C. albicans* in the host determine the infection trend, and the former affects the latter to some extent, but the latter is not always affected by the former^31^. The *atp16*Δ/Δ mutant exhibited selective growth defects in vitro, but could grow in the tissue grinders and serum that mimic the host environment (CFU value up to approximately 1/3 of that of WT) and remain persistent in mice with macrophages suppressed by clodronate without lethal infection. This suggests that the lack of pathogenicity displayed by the *atp16*Δ/Δ mutant is not due to growth defects, but to virulence defects. In this study, we found that δ subunit deletion downregulated virulence factors in vitro, and especially impaired filamentation ability after macrophage phagocytosis, again supporting that the reduced pathogenicity is mainly due to virulence defects. It has been reported that among 674 *C. albicans* gene-deleted mutants, 59% of these growth-defective mutants were competent for virulence, and 79% of virulence-defective mutants showed a normal growth in vitro, indicating that growth defects were not necessarily correlated to virulence^31^. In a previous study, we also found that *C. albicans* F_1_F_o_-ATP synthase i/j subunit deletion resulted in no growth defects in vitro, but significantly reduced pathogenicity^33^.

We found that δ subunit deletion decreased oxidative respiration and coupling function, impaired ATP production from OXPHOS, and downregulated the expression levels of mitochondrial genes and OXPHOS proteins, including F_1_F_o_-ATP synthase proton channel core forming subunits and dimer formation contributing subunits, which likely affected the process of F_1_F_o_-ATP synthase proton channel assembly and may contribute to alterations in inner membrane structure.

A key signal transduction pathway that regulates multiple virulence traits of *C. albicans* is the cAMP-PKA pathway^27,28^. We revealed that δ subunit deletion blocked the cAMP-PKA pathway to inhibit expression of multiple virulence factors (Supplementary Fig. 9). The activity of the cAMP-PKA pathway can be modulated by intracellular ATP^27,29^. There was no change in the intracellular ATP concentration after δ subunit deletion, but we found defects in intracellular FBP level, and the addition of FBP restored filamentation and upregulated Ras1-dependent and -independent cAMP-PKA pathways in *atp16*Δ/Δ, indicating that the δ subunit regulates the cAMP-PKA pathway through FBP. This conclusion is consistent with previous work^34^ showing that human FBP activates Ras by binding directly to Sos1 and dissociating the Ras/Sos1 complex. We further determined that δ subunit deletion weakened Pfk1 activity upstream of FBP. Pfk1 activity is mainly regulated by chemical modifications and conformations^35^. Pfk1 activity is diminished/enhanced by inhibited/enhanced phosphorylation respectively, during which phosphorylation maintains enzymatic activity by stabilizing its conformation, regulating the substrate F6P cooperativity and making it less susceptible to ATP inhibition^36^. In this paper, we further found that δ subunit deletion decreased Pfk1 phosphorylation (Supplementary Fig. 9) and enhanced its binding to the ATP analogue Cibacron Blue F3G-A. Cibacron Blue F3G-A binding results in a decrease in Pfk1 affinity to the substrate F6P^37^. These results suggest that δ subunit deletion reduces Pfk1 activity by decreasing its phosphorylation and causing conformation shifts to decrease its affinity to the substrate F6P. The mechanisms by which δ subunit deletion induces reduced Pfk1 phosphorylation and activity remain unknown, but we speculate that the deletion may affect Pfk1 activity by disrupting the mitochondrial membrane structure. In summary, δ subunit deletion reduces Pfk1 activity by interrupting its phosphorylation to cause conformation shifts, decreases downstream FBP, blocks cAMP-PKA pathways, and curtails the production of multiple virulence factors.

## Methods

### Strains and culture conditions

Strains used in this study are listed in Supplementary Table 3. For routine propagation, the strains were grown in yeast extract peptone dextrose (YPD) broth or on YPD agar. For the selection of nourseothricin-resistant strains, YPD with 200 μg/ml nourseothricin (YPD-Nou) was used^38^.

### Generation of the gene deletion mutant, complementation strain and overexpression strain

*C. albicans* SC5314 (WT) was used to generate the *atp16*Δ/Δ homozygous mutant (*atp16*Δ*::FRT*/*atp16*Δ*::FRT*), *atp16*Δ/*ATP16* complemented strain (*atp16*Δ*::FRT*/*ATP16*::*FRT*) and Atp16 O/E overexpression strain (Supplementary Table 3). A homozygous mutant of *ATP16* (*atp16*Δ/Δ) was constructed by using the *SAT1*-Flipper method^38,39^. Briefly, the construction of the deletion cassette was based on plasmid pSFS2, which was genetically cloned approximately 400 bp upstream and downstream of the *ATP16* ORF. The flanking upstream and downstream ranges of *ATP16* were amplified by PCR with primers ATP16–1 plus ATP16–2 and ATP16–3 plus ATP16–4, and introduced into the ApaI/XhoI and NotI/SacII sites of pSFS2, respectively. The resulting deletion cassette was linearized with SacII and used to transform into SC5314, with selection on YPD-Nou. Correct integration of the deletion cassette was verified by PCR amplification of genomic DNA with primers ATP16–5 plus ATP16–6 and ATP16–7 plus ATP16–8. Nourseothricin sensitivity was restored by inducing the expression of the Mal2p-FLP recombinase gene through growth on yeast extract-peptone-maltose medium. This is the heterozygous mutant of *ATP16* (*ATP16*/*atp16*Δ). This process was repeated to generate the second allele deletion of *atp16*Δ/Δ. The linearized deletion cassette was used to transform into *ATP16*/*atp16*Δ, with selection on YPD-Nou. Correct integration of the deletion cassette was verified by PCR amplification with primers ATP16–5 plus ATP16–6 and ATP16–7 plus ATP16–8, and *ATP16* ORF deletion was verified by PCR amplification with primers ATP16-F plus ATP16-R. Nourseothricin sensitivity was restored by inducing the expression of the Mal2p-FLP recombinase gene through growth on yeast extract-peptone-maltose medium. This is the homozygous mutant of *ATP16* (*atp16*Δ/Δ). For the complemented strain, one allele complementation of the mutant strain was performed with the complemented plasmid. The construction of the complemented cassette was based on plasmid pSFS2-down, which carries the approximately 400 bp downstream of the *ATP16* ORF. The entire *ATP16* ORF with flanking approximately 400 bp upstream and downstream was amplified by PCR and cloned into ApaI/XhoI sites of pSFS2-down. The resulting complemented cassette was linearized with SacII and used to transform into an *atp16*Δ/Δ homozygous mutant to generate the *atp16*Δ/*ATP16* complemented strain. For the overexpression strain, the entire *ATP16* gene with its native promoter was amplified by PCR with ATP16orf-F plus ATP16orf-R, cloned into XhoI/BglII sites of pADH1E2^40^ and transformed into SC5314 to generate the Atp16 O/E overexpression strain. Correct integration was verified by PCR amplification of genomic DNA with primers pADH1E2-ATP16-up-F plus pADH1E2-ATP16-up-R and pADH1E2-ATP16-down-F plus pADH1E2-ATP16-down-R. The primers used for gene deletion, complementation and overexpression in this study are listed in Supplementary Table 4. Schematic diagrams of the *atp16*Δ/Δ mutant, *atp16*Δ*/ATP16* complemented strain and Atp16 O/E overexpression strain are shown in Supplementary Figs. 10, 11 and 12. Confirmation of the *atp16*Δ/Δ mutant, *atp16*Δ*/ATP16* complemented strain and Atp16 O/E overexpression strain was performed via PCR and RT-qPCR analysis, as shown in Supplementary Figs. 13 and 14.

### In vivo virulence assay

Murine systemic candidiasis is a well-established model to study pathogenicity by *C. albicans*^41,42^. *C. albicans* strains were cultured in YPD broth at 30 °C for 12 h, washed with NS and resuspended in NS. Female BALB/c mice (8–10 weeks old) were housed in conditions with controlled temperature (20–26 °C), humidity (40–70%), and 12/12-hour dark/light cycle. Mice were intravenously infected with 5 × 10^5^ CFU of WT, 5 × 10^5^ CFU of *atp16*Δ/Δ, 5 × 10^5^ CFU of *atp16*Δ/*ATP16*, 5 × 10^5^ CFU of Atp16 O/E, or 5 × 10^6^ CFU *atp16*Δ/Δ or inoculated with 100 µl of NS as a control. Mouse survival was monitored and recorded daily. To investigate hepatic and renal function, three mice from each group were weighed and humanely euthanized at 1, 24, 48 and 72 h postinfection. Blood was collected with anticoagulated heparin (100 U administered intraperitoneally; Sigma-Aldrich) and centrifuged at 2000 × *g*, and the serum was frozen at −20 °C until analysis. Serum urea, creatine and alanine aminotransferase levels were determined by using an autoanalyser for a clinical chemistry assay (Beckman Coulter AU5800). *C. albicans* fungal burdens were assessed from various organs as described^43^. To reveal tissue damage, kidneys, brains, livers and spleens were aseptically removed from mice at 1, 24, 48 and 72 h postinfection, fixed in 10% neutral-buffered formalin, processed according to standard procedures, embedded in paraffin, and sectioned. Sections were stained with periodic acid-Schiff (PAS) and haematoxylin and eosin (HE). Stained slides were scanned using a Pannoramic slide scanner (Pannoramic 250/MIDI) and evaluated for their inflammation severity. Renal inflammation was scored as previously described^44^. For clodronate- or PBS-liposome-treatment experiments, mice were injected intraperitoneally with 100 µl of liposomes per 10 g of mouse weight both 24 h before and 24 h after intravenous infection with 2 × 10^5^ CFU of *C. albicans* WT or *atp16*Δ/Δ. Clodronate or PBS control liposomes were obtained from Vrije Universiteit Amsterdam and handled according to the manufacturer’s instructions. For S1 in vivo antifungal activity, mice were intravenously infected with 2 × 10^5^ CFU of WT as previously described. Treatment with vehicle (20% cyclodextrin) or S1 (50 or 100 mg/kg once daily) began 2 h before inoculation and continued for 7 days. All mouse infection experiments were approved by the ethics committee of Jinan University, Guangdong, China.

### MicroPET/CT and biodistribution assays

Three mice from each group were anaesthetized by intraperitoneal injection of 1% pentobarbital and tails were intravenously injected with [^18^F]FDG (Guangzhou HTA Isotope Medical Co. Ltd) (10–11 MBq) at 24, 48 and 72 h after infection with *C. albicans* (5 × 10^5^ CFU). Forty minutes after the injection, the mice were scanned with a small-animal PET/CT scanner (Inveon) for 20 min. All PET images were reconstructed with the maximum a posteriori (MAP) reconstruction algorithm in Inveon Acquisition Workspace (Siemens Medical Solutions Inc.) and analysed with Inveon Research Workspace 4.2 (Siemens Medical Solutions Inc.) after the coregistration of the PET/CT images. Regions of interest (ROIs) were drawn in the kidneys. The radioactivity density from image analysis is presented as the percent injected dose per gram (% ID/g). After the final imaging time point, the mice were sacrificed. The blood, brain, heart, lungs, liver, spleen, kidneys, stomach, colon and muscle were harvested for ex vivo biodistribution analysis. The radioactivity in each organ was measured with an automatic gamma counter (Perkin-Elmer) after which the organ weights were determined with a balance. The biodistribution is presented as a percent of the injected dose per gram (% ID/g)^45,46^.

### Growth assay

For the in vitro growth assay, *C. albicans* strains were inoculated into fresh YPD broth and grown with shaking at 30 °C overnight. Then, the *C. albicans* strains were inoculated in 100 ml of YPD or yeast extract peptone succinate (YPS) broth adjusted to an initial absorbance of 0.02 at 600 nm using a Varioskan LUX Multimode Microplate Reader (Thermo Scientific) and grown with shaking at 30 °C. At the indicated time points, samples were taken to measure the absorbance at 600 nm, and appropriate dilutions of each sample were plated on duplicate on YPD plates to determine the number of CFU^47^. For the ex vivo growth assay, the kidneys, livers, spleens, brains and hearts were removed aseptically from the mice. The organs were cut into small pieces by using sterile scissors and forceps. With a sterile tissue grinder, an even suspension of the tissue was prepared using sterile saline solution (1 g of tissue:2 ml of saline solution). The suspension was poured into a large, screw-capped test tube using small amounts of saline solution to rinse all particles from the grinder. The suspension was placed onto a sterile 6-well plate (Corning), and the homogenate was inoculated with *C. albicans*. At the indicated time points, samples were plated on YPD agar for 48 h to determine the CFU/ml.

### Metabolomics analysis

*C. albicans* strains were cultured in YNB + 2% glucose broth at 30 °C for 12 h, washed with PBS and pelleted. The metabolites in *C. albicans* cells were extracted first with 200 µl of H_2_O (4 °C), vortexed for 30 s, and incubated in liquid nitrogen for 1 min, which was followed by sonication for 10 min. This freeze-thaw cycle was repeated three times in total. Each sample was added to 800 µl of ACN:MeOH (1:1, v/v), vortexed for 30 s, sonicated for 20 min, and then incubated for 1 h at -20 °C for protein precipitation. Afterwards, the sample was centrifuged for 20 min at 14,000 × *g* and 4 °C. The supernatant was collected and vacuum dried. Before loading to the LC-MS/MS, the sample was resolved in 100 µl of acetonitrile-water (1:1 v/v) solution and centrifuged for 20 min at 14,000 × *g* and 4 °C, and then the supernatant was collected for analysis. Six LC-MS/MS experiments were performed with 6 biological replicates from each group. Each sample was separated on an Agilent 1290 HPLC system. Mobile phase A was a water solution with 25 mM ammonium acetate and 25 mM ammonium hydroxide (pH 9.75) and mobile phase B was acetonitrile. The sample was placed in a sample loader at 4 °C, the temperature of the column was 45 °C, the flow rate was 300 µl/min, and the volume of the sample was 2 µl. The liquid phase gradient was as follows: 0–1 min, B was maintained at 90%; 1–14 min, the percentage of B decreased linearly from 90% to 65%; 14–18 min, the concentration of B was maintained at 40%; 18–18.1 min, the percentage of B increased from 40 to 90% and 18.1–23 min, the concentration of B was kept at 90%. One QC run was inserted after every 6 sample runs to evaluate the stability and reproducibility of the system. An Agilent 5500 QqQ spectrometer (Agilent Technologies) was used for MS analysis. ESI source conditions were set as follows: source temperature, 450 °C; ion source gas 1 (Gas1), 45; ion source gas 2 (Gas2), 45; curtain gas (CUR), 30 and ion spray voltage floating (ISVF), −4500 V. MRM mode was selected to monitor the ion pairs. The original MRM raw data of the metabolites were processed with an MRM Analyser based on detection and peak area integration from the individual target metabolites.

### Proteomic analysis

*C. albicans* strains were cultured in YNB + 2% glucose broth at 30 °C for 12 h, washed with PBS and pelleted. *C. albicans* cells were ground in liquid nitrogen and then sonicated three times on ice using a high-intensity ultrasonic processor (Scientz) in lysis buffer (including 1% Triton X-100, 10 mM dithiothreitol, 1% protease inhibitor cocktail (Sigma-Aldrich), 50 µM PR-619, 3 µM TSA, 50 mM NAM and 2 mM EDTA). An equal volume of Tris-saturated phenol (pH 8.0) was added and followed by vortexing. After centrifugation, the upper phenol phase was collected. Proteins were precipitated by adding at least four volumes of ammonium sulfate-saturated methanol and incubating at −20 °C for 6 h. After centrifugation, the supernatant was discarded. The remaining precipitate was washed once with ice-cold methanol once followed by ice-cold acetone for three times. The protein was redissolved in 8 M urea. For digestion, the protein solution was reduced with 5 mM dithiothreitol for 30 min at 56 °C and alkylated with 11 mM iodoacetamide for 15 min at room temperature in the dark. The protein was then diluted by adding 100 mM TEAB to a urea concentration of less than 2 M. Finally, trypsin was added at a 1:50 trypsin-to-protein mass ratio for the first digestion overnight and a 1:100 trypsin-to-protein mass ratio for a second 4 h digestion. After trypsin digestion, the peptide was desalted with a Strata X C18 SPE column (Phenomenex) and vacuum-dried. The peptide was reconstituted in 0.5 M TEAB and processed according to the manufacturer’s protocol for the TMT kit/iTRAQ kit. Briefly, one unit of TMT/iTRAQ reagent was thawed and reconstituted in acetonitrile. This reagent was then incubated with the peptide mixtures were then incubated for 2 h at room temperature, pooled, desalted and dried by vacuum centrifugation. The tryptic peptides were fractionated by high-pH reverse-phase HPLC using a Thermo Betasil C18 column (5 µm particles, 10 mm ID, 250 mm length). Briefly, peptides were first separated with a gradient of 8% to 32% acetonitrile (pH 9.0) over 60 min into 60 fractions. Then, the peptides were combined into 6 fractions and dried by vacuum centrifugation. The tryptic peptides were dissolved in 0.1% formic acid (solvent A) and directly loaded onto a homemade reversed-phase analytical column. The gradient was as follows: an increase from 6 to 23% solvent B (0.1% formic acid in 98% acetonitrile) over 26 min; 23 to 35% B over 8 min; climbing to 80% B over 3 min; and then holding at 80% B for the last 3 min. The flow rate was held constant at 400 nl/min on an EASY-nLC 1000 UPLC system. The peptides were subjected to an NSI source followed by tandem mass spectrometry (MS/MS) (Thermo Scientific) coupled online to the UPLC. The electrospray voltage applied was 2.0 kV. The m/z scan range was 350–1800 for a full scan, and intact peptides were detected in the Orbitrap at a resolution of 70,000. Peptides were then selected for MS/MS using an NCE setting of 28, and the fragments were detected in the Orbitrap at a resolution of 17,500. A data-dependent procedure that alternated between one MS scan followed by 20 MS/MS scans with 15.0 s dynamic exclusion was utilized. The automatic gain control (AGC) was set at 5E4. The fixed first mass was set as 100 *m/z*. Three experiments were performed with 3 biological replicates from each group.

### Intracellular ATP content assay

*C. albicans* strains were cultured in YPD broth at 30 °C for 12 h, washed with PBS and resuspended in different culture media to investigate investigating intracellular ATP content. The intracellular ATP content was assessed by a BacTiter-Glo™ Microbial Cell Viability Assay (Promega) following the manufacturer’s instructions. Briefly, an aliquot of 2 × 10^6^ CFU or 2 × 10^5^ CFU from each strain was mixed with the same volume of BacTiter-GloTM luciferase reagent and incubated for 15 min at room temperature in the dark. Luminescence was measured by using a Varioskan LUX Multimode Microplate Reader (Thermo Scientific).

### OCR and ECAR assays

A Seahorse Xfe96 analyser (Agilent) was used to measure the OCR and ECAR as described^48^. Data were analysed using the Wave 2.6. In brief, exponentially grown *C. albicans* cells were seeded at a density of 5 × 10^5^ CFU per well in poly-L-lysine (0.03%) precoated Seahorse XF96 cell culture microplates (Agilent), centrifuged and incubated for 1 h at 30 °C to permit cell adhesion to the microtiter plates. The OCR was examined by sequential injections of DCCD (100 μM), FCCP (2 μM) and rotenone/antimycin A (0.5 μM). The indices of mitochondrial function were calculated as basal respiration and ATP synthesis. The ECAR was examined through sequential injections of glucose (10 mM), rotenone/antimycin A (0.5 μM) and 2-DG (50 mM). The indices of glycolytic function were calculated as glycolysis.

### ROS assay

ROS measurement was performed by using the oxidation-sensitive fluorescent dye 2′,7′-dichlorofluorescein diacetate (DCFDA) (Abcam) according to the manufacturer’s protocol. Briefly, 2 × 10^6^ CFU of exponentially grown *C. albicans* cells were stained with DCFDA (20 µg/ml) at 37 °C for 20 min in the dark, washed with PBS and analysed with a FACScan flow cytometer (Becton Dickinson) (Ex/Em of 595/488 nm). Data were analysed using the BD FACSDiva v8.0.1. Populations were gated to exclude debris and doublets (Supplementary Fig. 15a), and the mean fluorescence intensity value (Supplementary Table 5) was taken for each sample.

### ΔΨm assay

ΔΨm measurements were performed by using the mitochondrial membrane potential dye 5,6-dichloro-2-[(*E*)-3-(5,6-dichloro-1,3-diethyl-1,3-dihydro-2*H*-benzimidazol-2-ylidene)-1-prop-1-enyl]-1,3-diethyl-1*H*-benzimidazolium iodide (JC-1) (Abcam) according to the manufacturer’s protocol. Briefly, 2 × 10^6^ CFU of exponentially grown *C. albicans* cells were incubated with JC-1 (20 μM) at 37 °C for 30 min in the dark, washed with PBS and analysed with a FACScan flow cytometer (Becton Dickinson) (Ex/Em of 595/488 nm). Data were analysed using the BD FACSDiva v8.0.1. The populations were gated to exclude debris and doublets (Supplementary Fig. 15b), and the red/green mean fluorescent intensity value (Supplementary Table. 6) was taken for each sample.

### F_1_F_o_-ATPase activity assay

Cells were grown overnight at 30 °C in YPD medium, and mitochondria were isolated by using a Yeast Mitochondria Isolation Kit (Sigma-Aldrich) according to the manufacturer’s instructions. Mitochondria (50 µg) were prepared to measure F_1_F_o_-ATPase activity. The ATP hydrolysis reaction was initiated by adding 1 mM ATP and then phosphate release was monitored at an absorbance of 360 nm using an EnzChek Phosphate Assay Kit (Invitrogen) according to the manufacturer’s instructions. The average hydrolysis rates in a time period from 1 to 5 min after initiation were calculated and presented as the amount of phosphate (mmol) released per min.

### TEM assay

*C. albicans* strains were grown overnight at 30 °C in YPD medium, and transferred to culture in YNB + 2% glucose broth at 30 °C for 12 h, washed with PBS and pelleted, fixed overnight in 4% paraformaldehyde and 2.5% glutaraldehyde fixative, and stored at 4 °C until processing for electron microscopy. Cells were postfixed in 1% osmium tetroxide, dehydrated in a series of ethanol solutions (15-minute washes in 10, 25, 40, 55, 70, 85 and 100% ethanol) and embedded in epoxy resin as described previously^49^. Thin sections (70 nm) were prepared and examined by TEM (JEOL, JEM-1230).

### Glycolytic rate assay

*C. albicans* strains were cultured in YPD broth at 30 °C for 12 h, washed with PBS and resuspended in YNB + 2% glucose. *C. albicans* cells were incubated for 16 h and then the culture medium was collected for measurement of the glucose concentration by using a glucose (GO) assay kit (Sigma-Aldrich). Glucose consumption was defined as the difference in glucose concentration in the medium with or without cell incubation. Glucose consumption was normalized according to cell number (per 10^6^ CFU).

### Hk2, Pfk1, Pyk1, G6PD activity and NADPH level assays

Hk2 activity was measured using a colorimetric quantitative assay kit (GenMed) following the manufacturer’s instructions. In brief, total protein (30 µg) was added to reaction buffer containing 40 mM triethanolamine (pH 7.6), 8 mM MgCl_2_, 0.2 M glucose, 0.75 mM ATP, 1 U/ml glucose 6-phosphate dehydrogenase, and 1 mM NADP. The reactions started when the protein was added to the reaction buffer. NADH oxidation was detected by measuring the decrease in absorbance at 340 nm in a microplate reader by reading once every 30 s for 5 min. The difference between the absorbance at the fifth minute and the immediate absorbance measurement represented the Hk2 activity. Pfk1 activity was measured as previously described^50^. Briefly, the reaction was performed using either total protein (30 µg) or purified Pfk1 (5 µg) in 250 μl of reaction buffer containing 50 mM Tris-HCl (pH 7.2), 10 mM MgCl_2_, 100 mM KCl, 5 mM fructose-6-phosphate, 2 mM ATP, 0.2 mM NADH, 0.3 U/ml aldolase, 2 U/ml triosephosphate isomerase, and 0.5 U/ml α-glycerophosphate dehydrogenase. The reactions started when the protein was added to the medium. NADH oxidation was detected by measuring the absorbance decrease at 340 nm with a microplate reader. Pyk1 activity was measured using a pyruvate kinase assay kit (Abcam). G6PD activity was determined using a Glucose 6 Phosphate Dehydrogenase Assay kit (Sigma-Aldrich). NADPH levels were determined using the NADP+/NADPH Quantification kit (Sigma-Aldrich).

### RT-qPCR assay

Total RNA was extracted from *C. albicans* cells as previously described^51^. cDNA was synthesized using PrimeScript RT Master Mix (TaKaRa Biotechnology). All quantitative real-time PCR was performed in triplicate using SYBR Green qPCR SuperMix (Invitrogen) and the amplified product was monitored with a MiniOpticon Real-time PCR System (Bio-Rad). The PCR was carried out with a denaturation step at 95 °C for 2 min, followed by 40 cycles of amplification and quantification at 95 °C for 15 s, 60 °C for 32 s, and 72 °C for 15 s and extension at 72 °C for 1 min. 18 S rRNA was the housekeeping gene for normalization. Data were analysed using the Bio-Rad CFX manager 3.1. The results were analysed using the 2^−ΔΔCT^ method. The primers for the genes used in the RT-qPCR are shown in Supplementary Table 4.

### Filamentation assay

A 2 × 10^5^ CFU/ml suspension of each strain in YPD, 10% FBS, Spider, or Lee’s liquid media was added to the wells of a 12-well flat-bottomed presterilized microtiter plate followed by incubation at 37 °C for 2 h. The plates were visualized under an inverted microscope and photographed. The percentage of hyphal cells and the length of the hyphae were calculated from at least 100 or 20 cells from each strain, respectively. In addition, cells from each strain were serially diluted and spotted on YPD, 10% FBS, Spider, Lee’s and SLAD agar and incubated at 37 °C for 7 days. The colonies were photographed.

### Adhesion assay

Adhesion was observed visually^52^. Flat bottomed presterilized microtiter plates (24-well) were incubated in foetal bovine serum overnight at 37 °C. A suspension of 1 × 10^6^ CFU of *C. albicans* in Spider medium was added to the wells followed by incubation at 37 °C for 2 h. Nonadherent cells were removed by washing with PBS. Fresh Spider medium was added to the corresponding wells, and the plates were incubated at 37 °C for another 24 h. The wells were washed and photographed.

### Biofilm formation assay

The biofilms were observed by confocal laser scanning microscopy (CLSM)^52^. Briefly, all strains were grown in glass bottom cell culture dishes. After 24 h of incubation at 37 °C, the resulting biofilms were washed and stained with 25 µg/ml concanavalin A-Alexa Fluor 594 conjugate (Invitrogen) at 37 °C for 1 h. CLSM was performed with a Zeiss LSM 510 upright confocal microscope (Carl Zeiss) using a Zeiss Achroplan ×40, 0.8-W objective, and a He-Ne laser with an excitation wavelength of 543 nm. Moreover, biofilm activity was assessed with an XTT reduction assay according to previously described protocols^53,54^.

### *C. albicans*-macrophage co-culture assays

For the macrophage killing assay, RAW264.7 cells (ATCC TIB-71) were seeded in 96-well plates at a density of 1 × 10^5^ CFU/well and incubated overnight. *C. albicans* cells were added at an MOI of 3 and coincubated for 2, 4, 6, and 8 h. The cocultures were treated with 0.1% Triton X-100 for 2 min at each time point and then diluted and applied to YPD plates. The CFUs were counted after 48 h of incubation at 30 °C. The *C. albicans* survival rate was determined by comparison of fungi recovered in the absence of macrophages. *C. albicans*-induced macrophage damage was assessed by measuring the release of lactate dehydrogenase (LDH) using a nonradioactive cytotoxicity assay (Promega). RAW264.7 cells (1 × 10^5^ cells/well) and *C. albicans* (2 × 10^5^ CFU/well) were added to 96-well plates and cocultured for 6, 12, 24 and 48 h. At each time point, the plate was centrifuged at 250 × *g* for 5 min, and 50 μl of the supernatant was transferred to a new 96-well plate and mixed with 50 μl of substrate mixture for 30 min. The reaction was stopped with the addition of 50 μl of stop solution, and the absorbance at 490 nm was measured and recorded. For CLSM, RAW264.7 cells were seeded in 35 mm glass bottom dishes and stained with 500 nM MitoTracker Deep Red FM (Molecular Probes) for 30 min. *C. albicans* cells were labelled with 1.25 mM fluorescein isothiocyanate (Sigma-Aldrich) for 15 min at room temperature and washed three times to remove excess dye. FITC-labelled *C. albicans* were cocultured with macrophages at an MOI of 2 for 3 h. The cultures were washed twice with PBS, and images were acquired using CLSM (Carl Zeiss LSM880) under the set of E_x644_/E_m655_ (macrophages) and E_x488_/E_m525_ (*C. albicans*). Data were analysed using the ZEN 2.3.

### PKA activity, intracellular cAMP concentration and Ras1 activity assays

*C. albicans* cells in Spider medium plus 0.2% glucose were incubated at 37 °C for 12 h, collected and washed with PBS. Total protein was extracted by using a Fungal Protein Extraction Kit (BestBio) following the manufacturer’s instructions. PKA activity was measured using a colorimetric quantitative assay kit (GenMed) following the manufacturer’s instructions. In brief, total protein was added to buffer containing the substrate and then the absorbance at 340 nm was measured every 1 min for 5 min. The difference between the absorbance at the fifth minute and the immediate absorbance reading represents the activity. The intracellular cAMP concentration was assessed with a cAMP-Glo™ Assay kit (Promega) following the manufacturer’s instructions. Ras1 activity was assessed by utilizing the Active Ras Pull-Down and Detection Kit (Pierce) following the manufacturer’s instructions. In general, 200 µg of total protein was used for pull-down unless otherwise specified. Then, 12.5 µl of the pull-down samples containing active Ras1 or a total of 10 µg of total protein for the input control was diluted in SDS loading buffer, separated by SDS-PAGE, transferred to polyvinylidene difluoride (PVDF) with the Trans-Blot Turbo Transfer system (Bio-Rad), and detected with monoclonal anti-Ras clone 10 antibody (1:1000; Millipore), followed by secondary detection with goat anti-mouse antibody (1:10,000; EarthOx Life Sciences). As a control protein, GAPDH was detected with a GAPDH polyclonal antibody (1:5000; Bioworld) as described previously^55^. Densitometry analysis of Ras1 levels on Western blots was conducted with ImageJ.

### Intracellular FBP and pyruvate concentrations assays

The intracellular FBP and pyruvate concentrations were assessed with a Yeast Fructose-1,6-bisphosphate Colorimetric Quantitative Assay kit (GenMed) and a Yeast Pyruvate Colorimetric Quantitative Assay kit (GenMed), respectively. To determine the intracellular FBP concentration, total protein was added to reaction buffer containing 100 mM Tris (pH 7.4), 5 mM MgCl_2_, 2 mM ATP, 0.2 mM NADH, 3 U/ml triosephosphate isomerase, 0.5 U/mlglycerol-3-phosphate dehydrogenase, and 1 U/ml Aldolase. To determine the intracellular pyruvate concentration, total protein was added to reaction buffer containing 100 mM Tris (pH 7.4), 3 mM MgCl_2_, 2 U/ml D-LDH, and 0.2 mM NADH. Reactions were started when the protein was added to the reaction buffer. NADH oxidation was detected by measuring the decrease in absorbance at 340 nm in a microplate reader once every 30 s for 5 min. The difference between the absorbance at the fifth minute and the immediate absorbance represents the metabolite concentration.

### Pfk1 purification and phosphorylation assay

Pfk1 was purified with slight modifications to the described procedure^56^. Briefly, for polyethylene glycol-6000 (PEG 6000) precipitation, total protein was extracted from *C. albicans* cells and quantitatively precipitated by the addition of PEG 6000 to a final concentration of 4%. After 30 min, the suspension was centrifuged at 19721 × g for 30 min. The supernatant was collected and quantitatively precipitated by the addition of PEG 6000 to a final concentration of 14%. After 30 min, the suspension was centrifuged at 19721 × *g* for 30 min. The precipitate was collected and suspended in buffer A containing 50 mM potassium phosphate buffer (pH 7.0), 0.5 mM phenylmethylsulfonyl fluoride, and 5 mM EDTA. For affinity chromatography, a HiTrap Blue HP prepacked column (GE Health care) was equilibrated with buffer A until the baseline was achieved and the eluent pH and conductivity were stable. The protein sample was then applied to the column and equilibrated with buffer A until baseline was reached and the eluent pH and conductivity were stable. Elution began using buffer B containing 50 mM potassium phosphate buffer (pH 7.0), 0.5 mM phenylmethylsulfonyl fluoride, 10 mM MgSO_4_, and 5 mM ATP. For ion exchange chromatography, a RESOURCE Q prepacked column (GE Health care) was equilibrated with buffer A until the baseline was obtained and the eluent pH and conductivity were stable. The protein sample was then applied to the column and equilibrated with buffer A until baseline was achieved and the eluent pH and conductivity were stable. Elution began using 0.5 M NaCl solution. Then, the Pfk1 solution was filtered with an Amicon® Ultra-15 Centrifugal Filter (Merck Millipore). Purified Pfk1 phosphorylation levels were determined by immunoblotting with an anti-phosphorylated tyrosine (p-Tyr) antibody (1:1000; PTM BIO), an anti-phosphorylated threonine (p-Thr) antibody (1:100; Abcam) and an anti-phosphorylated serine (p-Ser) antibody (1:800; Abcam).

### Homology modelling and virtual screening

Homology modelling relied on the electron microscopy structure of F_1_F_o_-ATP synthase bound to oligomycin determined at 3.8 Å resolution (6CP3 in the Protein Data Bank) and conducted in MOE v2015.1001 software. The protonation state of the protein and the orientation of the hydrogens were optimized by LigX at a pH of 7 and a temperature of 300 K. Ten intermediate models were built, and the one that scored the best according to the GB/VI scoring function was selected as the template. For virtual screening, the binding pocket was identified by the Site Finder module, and the three-dimensional structures of the compounds from the Specs chemical libraries (https://specs.net/) were filtered by Opera’s lead-like filter in MOE. The remaining compounds were first ranked by high-throughput rigid docking with London dG scoring. Then, the compounds with docking scores lower than −10.0 (kcal/mol) were selected for flexible docking and ranked by London dG scoring. Finally, force field refinement was carried out on the top 10 poses followed by rescoring with GBVI/WSA dG. The top 100 ranked hits were selected and divided into structural clusters through fingerprint-based clustering and a diverse subset with 20 hits was finally identified. We bought 20 representative compounds including S1 from Specs (https://specs.net/) to investigate the antifungal activities.

### Data analysis and statistical analysis

All values in the paper are given as the means ± SD unless stated otherwise. All experiments were performed in at least three independent trials. The figures were generated, and statistical analysis was performed using GraphPad Prism software (version 8.0.1). Data were analysed by using two-tailed unpaired Student’s *t*-test, one-way ANOVA or two-way ANOVA, as indicated. For survival analyses, log-rank tests were performed. *P* < 0.05 was accepted as statistically significant except for proteomics analysis and metabolomics analysis, in which a fold change >1.5 was defined as statistically significant.

### Reporting summary

Further information on research design is available in the Nature Research Reporting Summary linked to this article.

## Supplementary information

Supplementary Information

Reporting Summary

## Data Availability

Proteomics data are deposited in the Pride database under the accession code PXD024729. Metabolomics data are deposited in the MetaboLights database under the accession code MTBLS3332. All raw data used to generate the main manuscript and supplementary figures can be found in the Source data file provided with this paper.

## Source data

Source data

## References

[1] Fungal disease frequency (GAFFI, 2017); Available at https://www.gaffi.org/why/fungal-disease-frequency/. The ‘95-95 by 2025’ Roadmap (GAFFI, 2015); Available at: http://www.gaffi.org/roadmap/.

[2] Denning DW (2016). Minimizing fungal disease deaths will allow the UNAIDS target of reducing annual AIDS deaths below 500000 by 2020 to be realized. Philos. Trans. R. Soc. Lond., B, Biol. Sci..

[3] Brown GD (2012). Hidden killers: human fungal infections. Sci. Transl. Med..

[4] Vander Heiden MG, Cantley LC, Thompson CB (2009). Understanding the Warburg effect: the metabolic requirements of cell proliferation. Science.

[5] Song J, Pfanner N, Becker T (2018). Assembling the mitochondrial ATP synthase. Proc. Natl Acad. Sci. USA.

[6] Duvezin-Caubet S, Caron M, Giraud MF, Velours J, di Rago JP (2003). The two rotor components of yeast mitochondrial ATP synthase are mechanically coupled by subunit delta. Proc. Natl Acad. Sci. USA.

[7] Gibbons C, Montgomery MG, Leslie AG, Walker JE (2000). The structure of the central stalk in bovine F(1)-ATPase at 2.4 A resolution. Nat. Struct. Biol..

[8] Fillingame RH (2000). Getting to the bottom of the F1-ATPase. Nat. Struct. Biol..

[9] Duvezin-Caubet S (2006). A “petite obligate” mutant of *Saccharomyces cerevisiae*: functional mtDNA is lethal in cells lacking the delta subunit of mitochondrial F1-ATPase. J. Biol. Chem..

[10] Tso GHW (2018). Experimental evolution of a fungal pathogen into a gut symbiont. Science.

[11] Xin H, Mohiuddin F, Tran J, Adams A, Eberle K (2019). Experimental mouse models of disseminated *Candida auris* Infection. mSphere.

[12] Lo HJ (1997). Nonfilamentous *C. albicans* mutants are avirulent. Cell.

[13] Zhuang H, Alavi A (2002). 18-fluorodeoxyglucose positron emission tomographic imaging in the detection and monitoring of infection and inflammation. Semin. Nucl. Med..

[14] Mochizuki T (2001). FDG uptake and glucose transporter subtype expressions in experimental tumor and inflammation models. J. Nucl. Med..

[15] Guo H, Bueler SA, Rubinstein JL (2017). Atomic model for the dimeric F_O_ region of mitochondrial ATP synthase. Science.

[16] Davies KM, Anselmi C, Wittig I, Faraldo-Gómez JD, Kühlbrandt W (2012). Structure of the yeast F1Fo-ATP synthase dimer and its role in shaping the mitochondrial cristae. Proc. Natl Acad. Sci. USA.

[17] Ostojic J (2013). The energetic state of mitochondria modulates complex III biogenesis through the ATP-dependent activity of Bcs1. Cell Metab..

[18] Classen JB, Mergner WJ, Costa M (1989). ATP hydrolysis by ischemic mitochondria. J. Cell. Physiol..

[19] Larsson C, Pahlman IL, Gustafsson L (2000). The importance of ATP as a regulator of glycolytic flux in *Saccharomyces cerevisiae*. Yeast.

[20] Calderone RA, Fonzi WA (2001). Virulence factors of *Candida albicans*. Trends Microbiol..

[21] Sudbery PE (2011). Growth of *Candida albicans* hyphae. Nat. Rev. Microbiol..

[22] Uwamahoro N (2014). The pathogen *Candida albicans* hijacks pyroptosis for escape from macrophages. mBio.

[23] Wellington M, Koselny K, Sutterwala FS, Krysan DJ (2014). *Candida albicans* triggers NLRP3-mediated pyroptosis in macrophages. Eukaryot. cell.

[24] Erwig LP, Gow NA (2016). Interactions of fungal pathogens with phagocytes. Nat. Rev. Microbiol..

[25] Netea MG, Joosten LA, van der Meer JW, Kullberg BJ, van de Veerdonk FL (2015). Immune defence against *Candida* fungal infections. Nat. Rev. Immunol..

[26] Shapiro RS, Robbins N, Cowen LE (2011). Regulatory circuitry governing fungal development, drug resistance, and disease. Microbiol. Mol. Biol. Rev..

[27] Grahl N (2015). Mitochondrial activity and Cyr1 are key regulators of Ras1 activation of *C. albicans* virulence pathways. PLoS Pathog..

[28] Huang G, Huang Q, Wei Y, Wang Y, Du H (2019). Multiple roles and diverse regulation of the Ras/cAMP/protein kinase A pathway in *Candida albicans*. Mol. Microbiol..

[29] Tao L (2017). Integration of the tricarboxylic acid (TCA) cycle with cAMP signaling and Sfl2 pathways in the regulation of CO_2_ sensing and hyphal development in *Candida albicans*. PLoS Genet..

[30] Rieg G (1999). Unanticipated heterogeneity in growth rate and virulence among *Candida albicans AAF1* null mutants. Infect. Immun..

[31] Noble SM, French S, Kohn LA, Chen V, Johnson AD (2010). Systematic screens of a *Candida albicans* homozygous deletion library decouple morphogenetic switching and pathogenicity. Nat. Genet..

[32] Skrzypek MS (2010). New tools at the Candida Genome Database: biochemical pathways and full-text literature search. Nucleic Acids Res..

[33] Zhao Y (2021). The fungal-specific subunit i/j of F_1_F_o_-ATP synthase stimulates the pathogenicity of *Candida albicans* independent of oxidative phosphorylation. Med. Mycol..

[34] Peeters K (2017). Fructose-1,6-bisphosphate couples glycolytic flux to activation of Ras. Nat. Commun..

[35] Sola-Penna M, Da Silva D, Coelho WS, Marinho-Carvalho MM, Zancan P (2010). Regulation of mammalian muscle type 6-phosphofructo-1-kinase and its implication for the control of the metabolism. IUBMB life.

[36] Cai GZ, Callaci TP, Luther MA, Lee JC (1997). Regulation of rabbit muscle phosphofructokinase by phosphorylation. Biophys. Chem..

[37] Nissler K, Kessler R, Schellenberger W, Hofmann E (1979). Effects of AMP and Cibacron blue F3G-A on the fructose 6-phosphate binding of yeast phosphofructokinase. Biochem. Biophys. Res. Commun..

[38] Reuss O, Vik A, Kolter R, Morschhauser J (2004). The SAT1 flipper, an optimized tool for gene disruption in *Candida albicans*. Gene.

[39] Sasse C, Morschhauser J (2012). Gene deletion in *Candida albicans* wild-type strains using the SAT1-flipping strategy. Methods Mol. Biol..

[40] Reuss O, Morschhauser J (2006). A family of oligopeptide transporters is required for growth of *Candida albicans* on proteins. Mol. Microbiol..

[41] de Repentigny L (2004). Animal models in the analysis of *Candida* host-pathogen interactions. Curr. Opin. Biotechnol..

[42] Spellberg B, Ibrahim AS, Edwards JE, Filler SG (2005). Mice with disseminated candidiasis die of progressive sepsis. J. Infect. Dis..

[43] Wirnsberger G (2014). Jagunal homolog 1 is a critical regulator of neutrophil function in fungal host defense. Nat. Genet..

[44] Wirnsberger G (2016). Inhibition of CBLB protects from lethal *Candida albicans* sepsis. Nat. Med..

[45] Rolle AM (2016). ImmunoPET/MR imaging allows specific detection of *Aspergillus fumigatus* lung infection in vivo. Proc. Natl Acad. Sci. USA.

[46] Seo JW (2020). Positron emission tomography imaging of novel AAV capsids maps rapid brain accumulation. Nat. Commun..

[47] Chauhan N (2005). Virulence and karyotype analyses of rad52 mutants of *Candida albicans*: regeneration of a truncated chromosome of a reintegrant strain (rad52/RAD52) in the host. Infect. Immun..

[48] Lamprecht DA (2016). Turning the respiratory flexibility of *Mycobacterium tuberculosis* against itself. Nat. Commun..

[49] Deng J (2018). FUS interacts with ATP synthase beta subunit and induces mitochondrial unfolded protein response in cellular and animal models. Proc. Natl Acad. Sci. USA.

[50] Hofmann E, Kopperschlager G (1982). Phosphofructokinase from yeast. Meth. Enzymol..

[51] Ruben S (2020). Ahr1 and Tup1 contribute to the transcriptional control of virulence-associated genes in *Candida albicans*. mBio.

[52] Nobile CJ (2006). Critical role of Bcr1-dependent adhesins in *C. albicans* biofilm formation in vitro and in vivo. PLoS Pathog..

[53] Pierce CG (2008). A simple and reproducible 96-well plate-based method for the formation of fungal biofilms and its application to antifungal susceptibility testing. Nat. Protoc..

[54] Krom BP, Willems HM (2016). In vitro models for *Candida* biofilm development. Methods Mol. Biol..

[55] Wu SY (2019). *Candida albicans* triggers NADPH oxidase-independent neutrophil extracellular traps through dectin-2. PLoS Pathog..

[56] Kopperschläger G, Johansson G (1982). Affinity partitioning with polymer-bound Cibacron blue F3G-A for rapid, large-scale purification of phosphofructokinase from Baker’s yeast. Anal. Biochem..

