# Supplementary material for: The δ subunit of F1Fo-ATP synthase is required for pathogenicity of Candida albicans
Source: Nat Commun. 2021 Oct 15;12:6041. doi: 10.1038/s41467-021-26313-9 (PMC8519961; doi:10.1038/s41467-021-26313-9)
Supplement: Supplementary file 1 — Supplementary Information [file 41467_2021_26313_MOESM1_ESM.pdf]

Supplementary Information

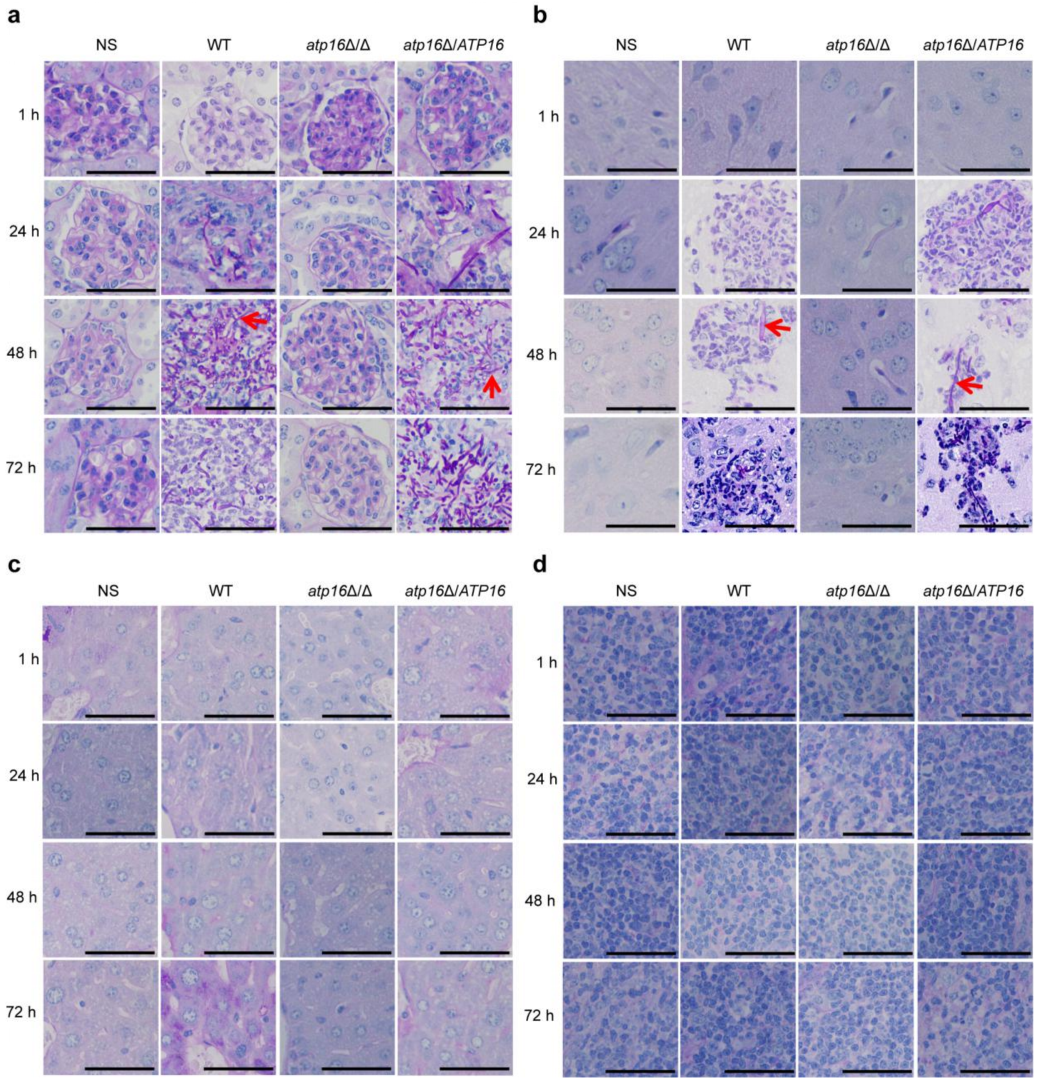

Supplementary Fig. 1 | Representative images of PAS-stained main organs.

Kidney, brain, liver and spleen sections of mice (*n* = 3) at 1, 24, 48, and 72 h after intravenous injection with NS and infection with WT, *atp16*Δ/Δ, and *atp16*Δ/*ATP16* (5×10^5 CFU per mice). **a**, Kidneys. *atp16*Δ/Δ displayed similar results to NS control. WT and *atp16*Δ/*ATP16* showed *C. albicans* filamentation at 1 h after infection, and the number of hyphae continued to increase throughout the entire course of the experiment. *C. albicans* colocalized with phagocytes to form abscesses at 24, 48, and 72 h after infection. **b**, Brains. *atp16*Δ/Δ displayed similar results to NS control. WT and *atp16*Δ/*ATP16* showed *C. albicans* filamentation at 24, 48, and 72 h after infection. *C. albicans* colocalized with phagocytes to form small scattered multifocal abscesses. **c** and **d**, Livers and spleens. *C. albicans* filamentation was not observed at any time point after infection; rather, phagocytes accumulated in large numbers but did not organize into abscesses in NS, WT, *atp16*Δ/Δ and *atp16*Δ/*ATP16.* In **a**, **b**, **c** and **d**, one representative experiment out of three independent experiments is shown. Arrowheads indicate fungal hyphae. Magnification × 400. Scale bar is 50 μM.

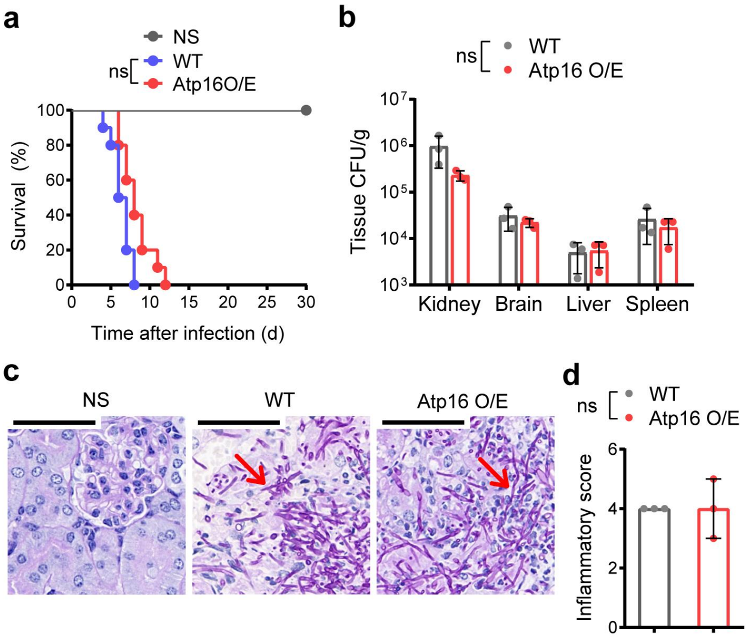

Supplementary Fig. 2 | Overexpression of the δ subunit does not enhance lethal *C. albicans* infection.

a, Survival curves of mice (*n* = 10) after intravenous infection with *C. albicans* Atp16 O/E (5 × 10^5 CFU per mice). **b**, *C. albicans* fungal burdens (CFU per g tissue) in organs (*n* = 3) 48 h after systemic infection with Atp16 O/E (5 × 10^5 CFU per mice). **c** and **d**, Representative images of PAS-stained kidney sections of mice (*n* = 3) (**c**) and combined inflammatory score based on renal immune cell infiltration (inflammation) and tissue destruction (*n* = 3) (**d**) 48 h after intravenous infection with Atp16 O/E (5 × 10^5 CFU per mice). Magnification × 400. Scale bar is 50 μM. In **a**, **b**, **c** and **d**, there were no significant differences in the phenotypes of Atp16 O/E compared with that of WT. In **c**, one representative experiment out of three independent experiments is shown. In **a**, **b** and **d**, data are expressed as the mean ± SD. ns, not significant; by log-rank test (**a**), or two-tailed unpaired Student's *t*-test (**b**, **d**).

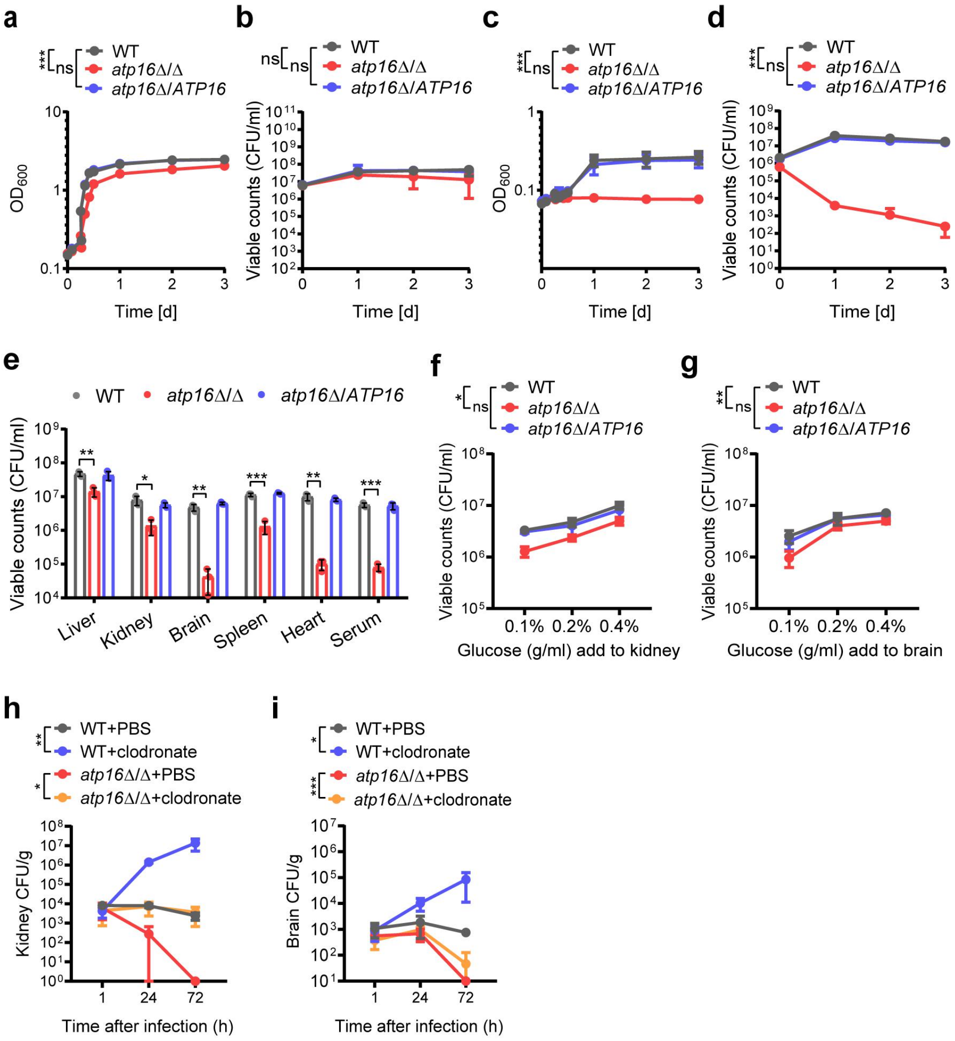

Supplementary Fig. 3 | Deletion of the δ subunit does not lead to remarkable *C. albicans* growth defects.

a-**d**, The growth of WT, *atp16*Δ/Δ and *atp16*Δ/*ATP16* in YPD (**a**, **b**) and YPS (**c**, **d**) media over time determined by cell density (OD_{600}) (**a**, **c**) and viable cell counts (CFU/ml) (**b**, **d**). **e**-**g**, The growth of WT, *atp16*Δ/Δ and *atp16*Δ/*ATP16* in liver, kidney, brain, spleen, and heart fresh tissue homogenates (500 mg/ml, without inactivation) and serum (100%, without inactivation) (**e**) and in kidney (**f**) and brain (**g**) homogenates (500 mg/ml, without inactivation) supplemented with 0.1%, 0.2%, and 0.4% glucose for 24 h determined by viable cell counts (CFU/ml). The initial density of *C. albicans* was $5×10^3$ CFU/ml. **h** and **i**, *C. albicans* fungal burdens (CFU per g tissue) in kidneys (**h**) and brains (**i**) of BALB/c mice that were injected with PBS liposomes (n = 3) or clodronate liposomes (n = 3) 24 h before and 24 h after intravenous infection with WT and *atp16*Δ/Δ ($2 × 10^5$ CFU per mice). In **a**, **b**, **c**, **d**, **e**, **f**, **g**, **h** and **i**, data are expressed as the mean ± SD of three independent experiments. *$P < 0.05$, **$P < 0.01$, ***$P < 0.001$; ns, not significant; by two-way ANOVA (**a**, **b**, **c**, **d**, **f**, **g**, **h** and **i**), or two-tailed unpaired Student's *t*-test (**e**).

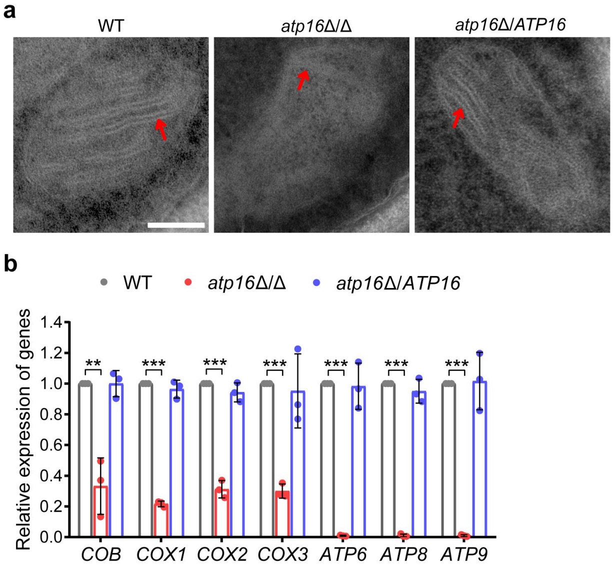

Supplementary Fig. 4 | The role of the δ subunit in the mitochondrial morphology and mRNA expression levels of mitochondrial genes.

a, The mitochondrial morphology of WT, *atp16Δ/Δ* and *atp16Δ/ATP16* cultured in YNB + 2% glucose medium for 12 h were observed by TEM. Arrowheads indicate the mitochondrial cristae. Magnification × 23000. Scale bar is 0.2 μM. **b**, The mRNA expression levels of the mitochondrial genes *COB* (encoded complex IV subunit 3), *COX1*, *COX2*, *COX3* (encoded complex IV subunits 1, 2, 3), *ATP6*, *ATP8*, and *ATP9* (encoded F_1F_o-ATP synthase subunits a, 8, c) in WT, *atp16Δ/Δ* and *atp16Δ/ATP16* were assessed by RT–qPCR. In **b**, Data are expressed as the mean ± SD of three independent experiments. ***P* < 0.01, ****P* < 0.001; by two-tailed unpaired Student's *t*-test.

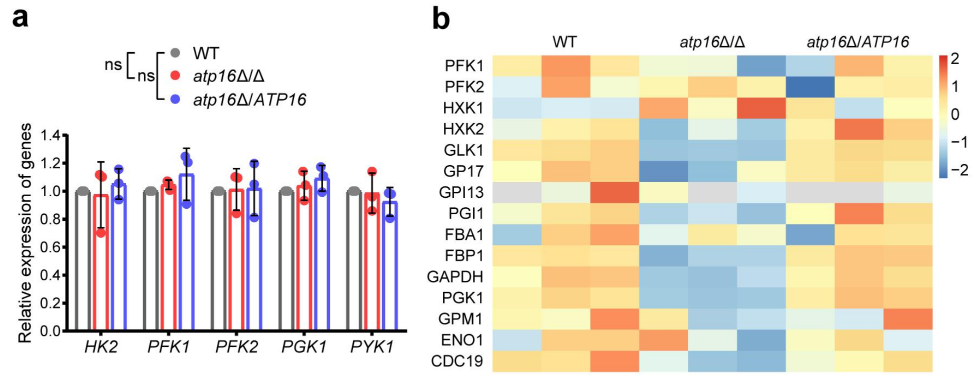

Supplementary Fig. 5 | The role of the δ subunit in the mRNA and protein expression levels of key enzymes in glycolysis.

a, Relative mRNA expression levels of glycolytic enzymes in WT, *atp16Δ/Δ* and *atp16Δ/ATP16* were assessed by RT–qPCR. **b**, The expression levels of proteins involved in glycolysis were assessed by proteomics analysis. The three columns for each strain represent three experiments performed with 3 biological replicates. In **a**, data are expressed as the mean ± SD of three independent experiments. ns, not significant; two-tailed unpaired Student's *t*-test.

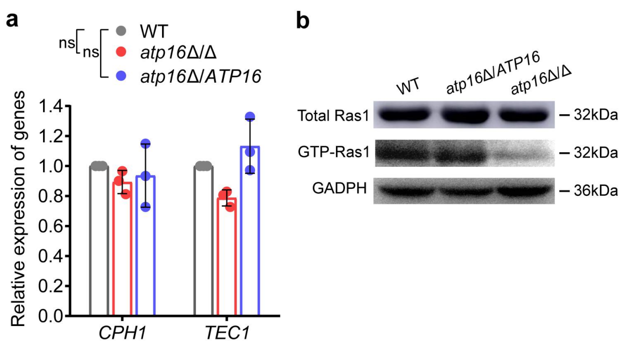

Supplementary Fig. 6 | The role of the δ subunit in the mRNA expression levels of the MAPK pathway transcription factors and activation of Ras1.

a, Relative mRNA expression levels of the MAPK pathway transcription factors Cph1 and Tec1 in WT, *atp16*Δ/Δ and *atp16*Δ/*ATP16* were assessed by RT–qPCR. **b**, Total Ras1 protein and GTP-Ras1 fraction in WT, *atp16*Δ/Δ and *atp16*Δ/*ATP16* were assessed by immunoprecipitation. GAPDH blots were shown as loading control. In **a**, data are expressed as the mean ± SD of three independent experiments. ns, not significant; by two-tailed unpaired Student's *t*-test. In **b**, one representative experiment out of three independent experiments is shown.

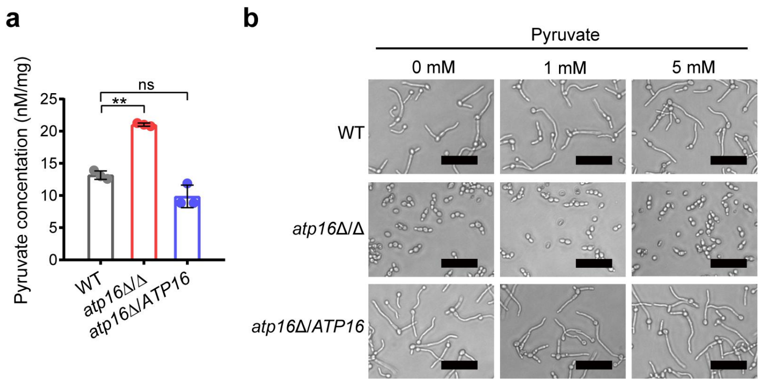

Supplementary Fig. 7 | The effects of pyruvate on *C. albicans* hyphal formation.

a, Intracellular pyruvate concentration per mg protein in WT, *atp16*Δ/Δ and *atp16*Δ/*ATP16*. **b**, Hyphae formation of WT, *atp16*Δ/Δ and *atp16*Δ/*ATP16* in the presence of exogenous pyruvate (0, 1, 5 mM) in Spider medium after incubation at 37 °C for 3.5 h. Magnification × 400. Scale bar is 50 μM. In **a**, data are expressed as the mean ± SD of three independent experiments. ***P* = 0.0037; ns, not significant; by two-tailed unpaired Student's *t*-test. In **b**, one representative experiment out of three independent experiments is shown.

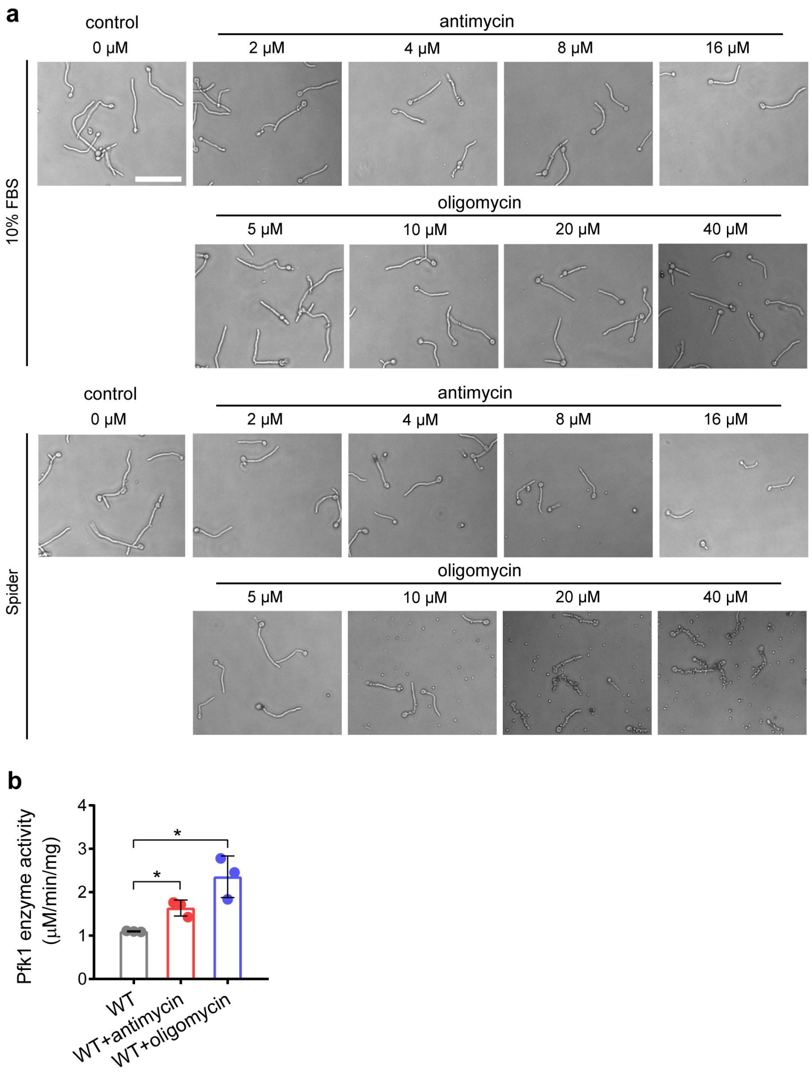

Supplementary Fig. 8 | The effects of antimycin/oligomycin on *C. albicans* hyphal formation and Pfk1 activity.

a, Hyphae formation of WT in the presence of the oxidation respiratory chain inhibitor antimycin (0, 2, 4, 8,16 μM) and F_1F_o-ATP synthase inhibitor oligomycin (0, 5,10, 20, 40 μM) after cultured in 10% FBS and Spider media at 37 °C for 3.5 h. Magnification × 400. Scale bar is 50 μM. **b**, The activity of Pfk1 in WT in the presence of the oxidation respiratory chain inhibitor antimycin (8 μM) and F_1F_o-ATP synthase inhibitor oligomycin (20 μM) after cultured in YPD medium at 30 °C for 12 h. In **b**, Data are expressed as the mean ± SD of three independent experiments. *P < 0.05; by two-tailed unpaired Student's t-test.

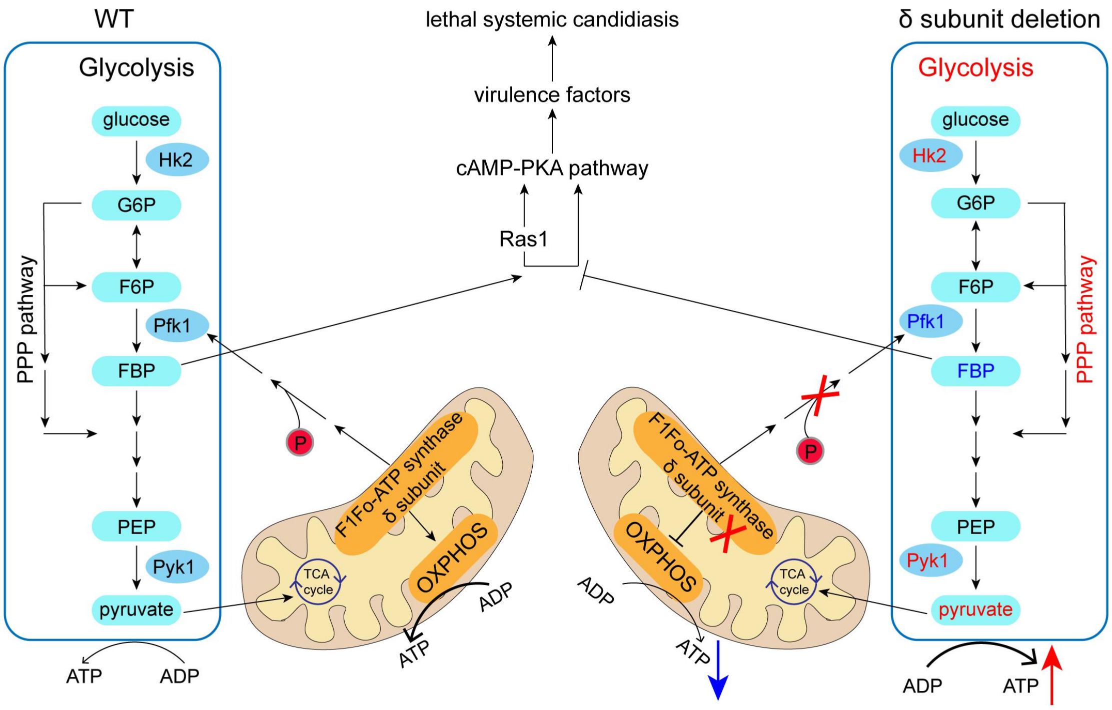

Supplementary Fig. 9 | Diagram illustrating that δ subunit deletion failed to cause lethal systemic candidiasis by regulating FBP-mediated cAMP-PKA signalling dependent virulence traits.

Compared with WT (**left**), δ subunit deletion interrupted Pfk1 phosphorylation, reduced enzymatic activity, decreased the downstream FBP level, blocked Ras1-dependent and -independent cAMP-PKA pathways, curtailed virulence factors, and finally protected mice from fatal infection (**right**). Red font and arrowhead represent increases, and blue font and arrowhead represent decreases.

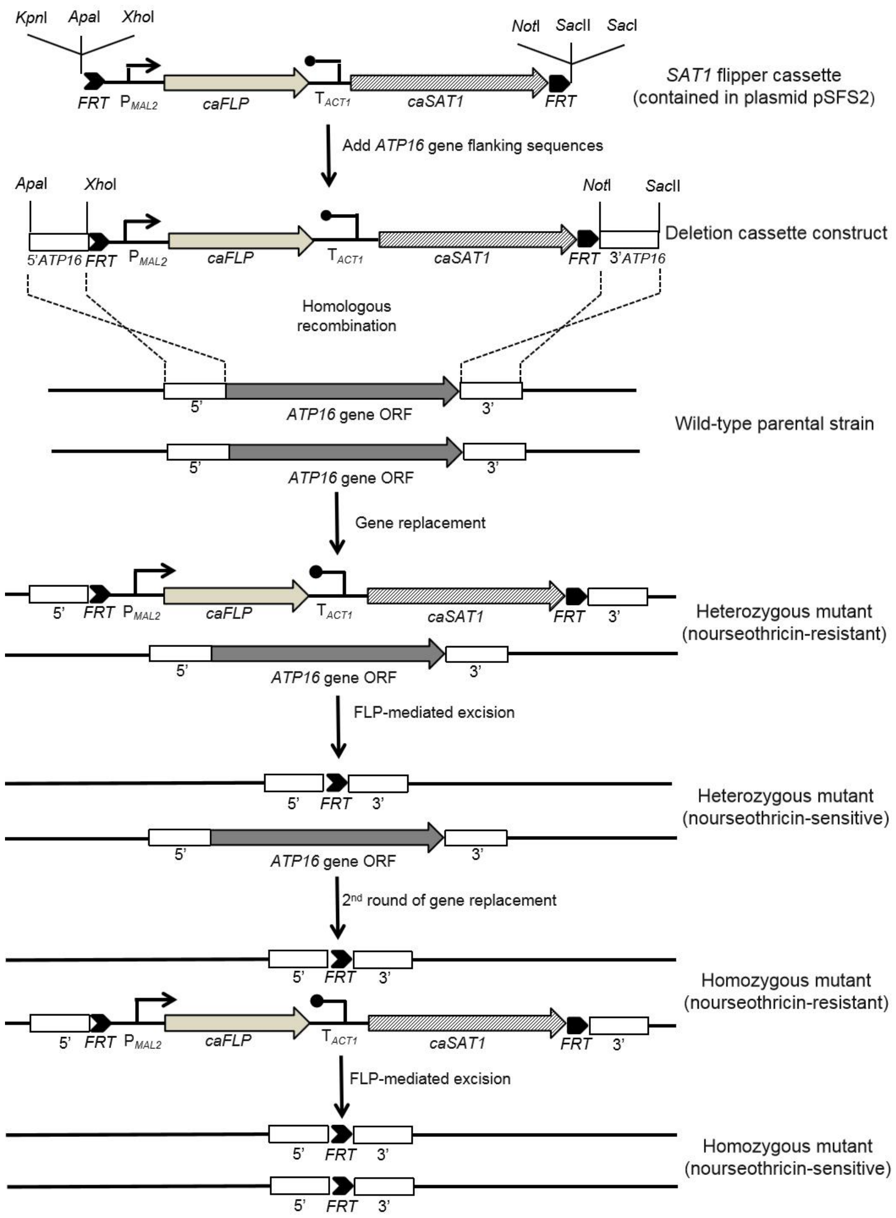

Supplementary Fig. 10 | Schematic diagram of the *ATP16* gene disruption by the *SAT1*-Flipper method.

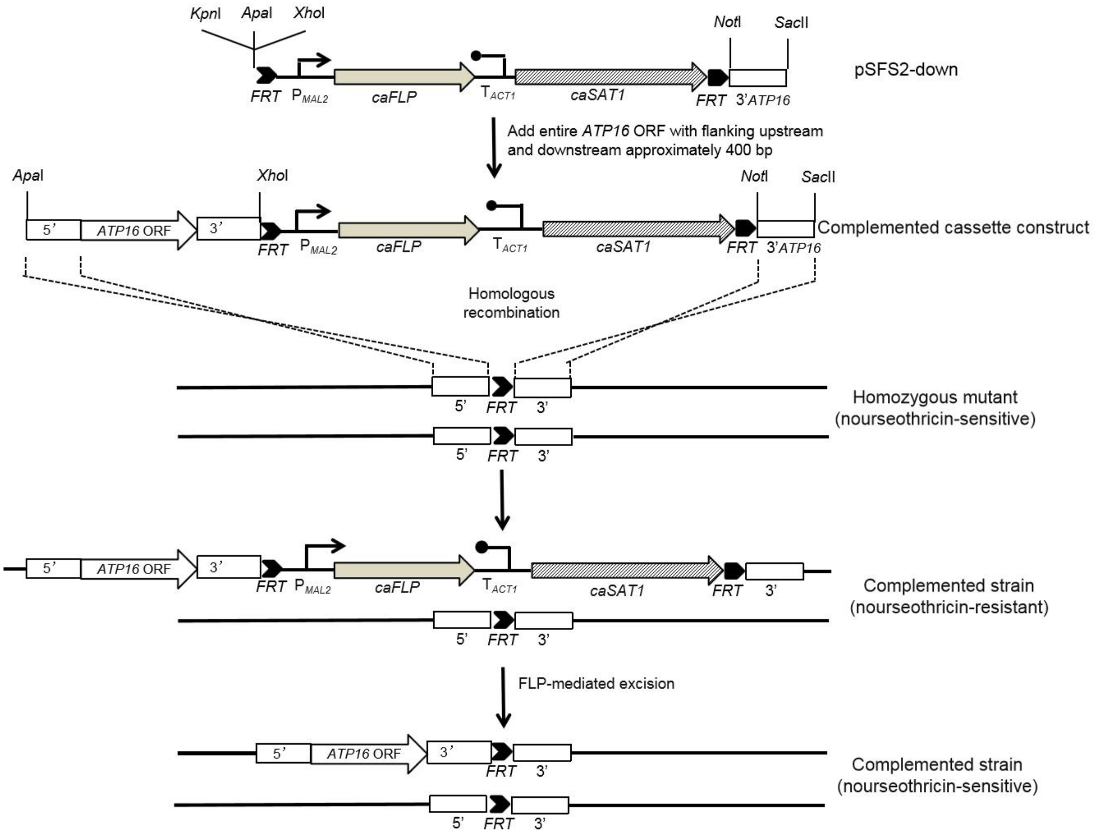

Supplementary Fig. 11 | Schematic diagram of the intact *ATP16* allele complemented into the *ATP16* homozygous mutant.

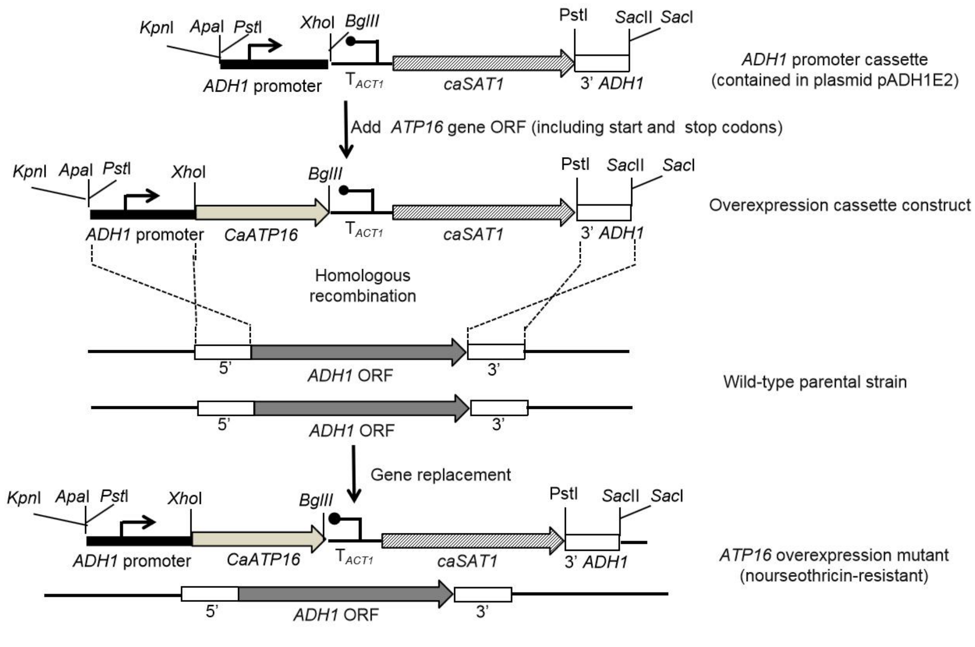

Supplementary Fig. 12 | Schematic diagram of the *ATP16* overexpression strain obtained by introducing an *ATP16* gene ORF to SC5314.

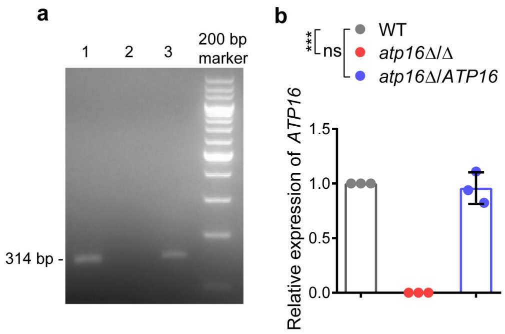

Supplementary Fig. 13 | Confirmation of *ATP16* gene deletion and complementation by PCR and RT─qPCR analysis.

a, The *ATP16* gene was PCR-amplified from the genomic DNA of the WT, *atp16*Δ/Δ and *atp16*Δ/*ATP16* with primers ATP16-F+ATP16-R. 1, WT was amplified stripe at 314 bp; 2, *atp16*Δ/Δ was without an amplified stripe; 3, *atp16*Δ/*ATP16* amplified stripe at 314 bp. **b**, RT─qPCR analysis of the mRNA level in *ATP16* from the three strains was performed. The relative mRNA level of *ATP16* in *atp16*Δ/Δ showed a 0.00015-fold change compared with WT; the relative mRNA level of *ATP16* in *atp16*Δ/*ATP16* showed a 0.47-fold change compared with WT. In **b**, data are expressed as the mean ± SD of three independent experiments. ****P* < 0.001; ns, not significant; by two-tailed unpaired Student's *t*-test.

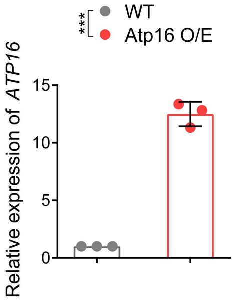

Supplementary Fig. 14 | Confirmation of *ATP16* gene overexpression by RT─qPCR analysis.

The relative mRNA level of *ATP16* in Atp16 O/E was approximately 12-fold different from that in WT. Data are expressed as the mean ± SD of three independent experiments. ****P* < 0.001; by two-tailed unpaired Student's *t*-test.

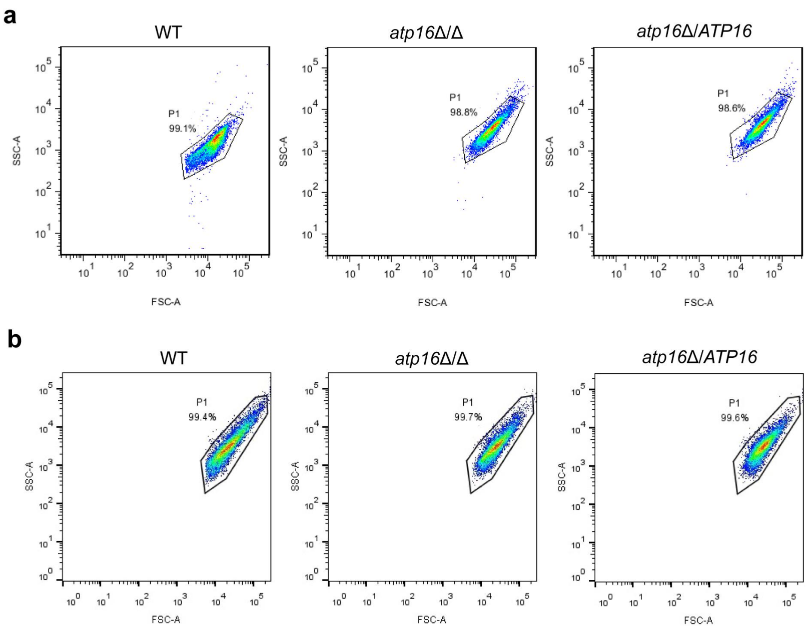

Supplementary Fig. 15 | Summary of the gating strategy from the ROS assay and ΔΨm assay.

Pseudocoloured density plots displaying the side-scatter and forward-scatter data for WT, *atp16*Δ/Δ and *atp16*Δ/*ATP16* cells when run on the FACScan flow cytometer and analysed with the FACSDiva Software. Gates displayed in black are the gates that were applied to all samples for data shown in Fig. 3e (**a**) and Fig. 3f (**b**).

Supplementary Table 1. Summary of pathogenicity-related proteins of *C. albicans* as determined in a murine model of candidiasis.

Homology with common pathogenic fungi	Non-homology with *Homo sapiens* and gut flora	Essentiality in lethal infection	Gene name	Protein accession	Protein name	Function	Biological process	Virulence in a disseminated candidiasis model	Reference
Yes	Yes	Yes	ATP16	A0A1D8PUD2	F1Fo-ATP synthase subunit delta	produces ATP from ADP	ATP synthesis coupled proton transport	avirulent	this study
Yes	Yes	Yes	FAS2	A0A1D8PK65	Fatty acid synthase subunit alpha	fatty-acid synthesis	Alpha subunit of fatty-acid synthase	avirulent	Zhao XJ, et al., Infect Immun, 1997; 65:829.
Yes	Yes	Yes	RFG1	Q5A220	Repressor of filamentous growth 1	filamentous, hyphal growth	transcriptional regulator	avirulent	Kadosh D, Johnson AD. Mol Cell Biol, 2001; 21: 2496.
Yes	Yes	Yes	VMA6	A0A1D8PKX3	V-type proton ATPase subunit	acidifying a variety of intracellular compartments	vacuolar proton-transporting V-type ATPase complex assembly	avirulent	Jia C, et al., Fungal Genet Biol, 2018; 114:1.
Yes	Yes	Yes	VMA7	A0A1D8PH17	V-type proton ATPase subunit F	vacuole acidification	putative vacuolar H+-ATPase (V-ATPase) subunit	avirulent	Poltermann S, et al., Microbiology, 2005; 151: 1645.
Yes	Yes	Yes	TPS1	Q92410	Alpha,alpha-trehalose-phosphate synthase	stress response, hyphal growth	trehalose-6-phosphate synthase	avirulent	Zaragoza O, et al., J Bacteriol, 1998; 180: 3809.
No	Yes	Yes	ACE2	Q59RR0	Cell wall transcription factor ACE2	morphogenesis	transcription activator	avirulent	Kelly MT, et al., Mol Microbiol, 2004; 53: 969.
No	Yes	Yes	ARG2	Q59MB6	Amino-acid acetyltransferase, mitochondrial, EC	N-acetylglutamate synthase involved in arginine biosynthesis	arginine biosynthetic process	avirulent	Epp E, et al., Mol Microbiol, 2010; 75: 1182.
No	Yes	Yes	ARV1	Q5ANH2	Protein ARV1	Mediator of sterol homeostasis	intracellular sterol transport	avirulent	Gallo-Ebert C, et al., Fungal Genet Biol, 2012; 49: 101.
No	Yes	Yes	CAP1 (SRV2)	Q5AJU7	AP-1-like transcription factor CAP1	cAMP-mediated signaling	Adenylate cyclase-associated protein	avirulent	Bahn YS, Sundstrom P. J Bacteriol, 2001; 183: 3211.
No	Yes	Yes	CDC35	A0A1D8PR83	Adenylate cyclase	cAMP-mediated signaling	adenylyl cyclase	avirulent	Rocha CR, et al., Mol Biol Cell, 2001; 12: 3631.

No	Yes	Yes	CRK1	Q9Y7W4	Serine/threonine-protein kinase BUR1	hyphal growth, pathogenesis	Protein kinase of the Cdc2 subfamily	avirulent	Chen J, et al., Mol Cell Biol, 2000; 20: 8696.
No	Yes	Yes	ECM33	Q5AGC4	Cell surface GPI-anchored protein ECM33	cell wall	cell surface GPI protein	avirulent	Martinez-Lopez R, et al., Microbiology, 2004; 150: 3341.
No	Yes	Yes	EDC3	A0A1D8PM93	Enhancer of mRNA-decapping protein 3	filamentous growth	cytoplasmic mRNA processing body assembly	avirulent	Jeong J, et al., Fungal Genet Biol, 2016; 97: 18.
No	Yes	Yes	FLO8	Q59QW5	Transcriptional regulator of filamentous growth FLO8	hyphal formation	transcription factor	avirulent	Cao F, et al., Mol Biol Cell, 2006; 17: 295.
No	Yes	Yes	FTR1	A0A1D8PFV0	High-affinity iron permease	iron transport	High-affinity iron permease	avirulent	Ramanan N, Wang Y. Science, 2000; 288: 1062.
No	Yes	Yes	GCN5	Q59PZ5	Histone acetyltransferase	Catalytic activity	pathogenesis	avirulent	Chang P, et al., Fungal Genet Biol, 2015; 81:132.
No	Yes	Yes	GOA1	Q5A672	GOA1 orf19.3818, CAALFM_C404640CA	oxidative stress	Protein required for respiratory growth	avirulent	Bambach A, et al ., Eukaryot Cell, 2009; 8: 1706.
No	Yes	Yes	LIP8	Q9P8V9	Lipase 8	Catalytic activity	pathogenesis	avirulent	Gácser A, Stehr F. Infect Immun, 2007; 75: 4710.
No	Yes	Yes	MCU1	Q5AKZ4	Mcu1p	carbon utilization	pathogenesis	avirulent	Guan G, et al., Fungal Genet Biol, 2015; 81: 150.
No	Yes	Yes	MGM1	Q5AFB7	Dynamin-related GTPase	GTPase activity	membrane fusion	avirulent	Liang C, et al., Fungal Genet Biol, 2018; 120: 42.
No	Yes	Yes	NAG5	Q59RW5	N-acetylglucosamine kinase 1, GlcNAc kinase 1	N-acetylglucosamine catabolic process	N-acetylglucosamine (GlcNAc) kinase	avirulent	Yamada-Okabe T, et al., Eur J Biochem, 2001; 268: 2498.
No	Yes	Yes	NDT80	Q5ACU9	Transcription factor	filamentous, hyphal growth	transcription factor	avirulent	Sellam A, et al., Eukaryot Cell, 2010; 9: 634.
No	Yes	Yes	NGG1	Q5AI28	Histone acetyltransferase	histone acetyltransferase activity	histone H3 acetylation	avirulent	Li D, et al., Future Microbiol, 2017; 12: 1497.
No	Yes	Yes	NOT4	A0A1D8PSH4	CCR4-NOT core ubiquitin-protein ligase subunit	filamentous growth, pathogenesis	Putative E3 ubiquitin-protein ligase	avirulent	Krueger KE, et al., Microbiology, 2004; 150: 229.
No	Yes	Yes	RTT109	Q5AAJ8	Histone acetyltransferase RTT109	histone acetylation, pathogenesis	histone acetyltransferase	avirulent	Lopes da Rosa J, et al., Proc Natl Acad Sci USA, 2010; 107: 1594.
No	Yes	Yes	SIT1	A0A1D8PEJ7	Basic amino acid transporter	ferrichrome transport	Transporter of ferrichrome siderophores	avirulent	Heymann P, et al., Infect Immun, 2002; 70: 5246.

No	Yes	Yes	SSK1	Q5AKU6	Oxidative stress response two-component system protein SSK1	cell wall organization, filamentous growth	Response regulator of two-component system	avirulent	Calera JA, et al., Infect Immun, 2000; 68: 518.
No	Yes	Yes	SWI1	Q59UR3	Swi1p	adhesion,hyphal growth	Protein involved in transcription regulation	avirulent	Mao X, et al., FEBS Letts, 2006; 580: 2615.
No	Yes	Yes	TUP1	P0CY34	Transcriptional repressor TUP1	Represses transcription	pathogenesis	avirulent	Murad AM, et al., EMBO J, 2001; 20: 4742.
No	Yes	Yes	VMA5	Q5A2U9	V-type proton ATPase subunit C	catalytic activity	Hydrogen ion transport	avirulent	Zhang K, et al., Future Microbiol, 2017; 12: 1147.
No	Yes	Yes	VPH1	Q59R99	V-type proton ATPase subunit a	catalyzes the translocation of protons across the membranes	vacuolar acidification	avirulent	Patenaude C, et al., J Biol Chem, 2013; 288: 26256.
No	Yes	Yes	VPS34	A0A1D8PDV7	Phosphatidylinositol 3-kinase VPS34	autophagy, endocytosis	phosphatidylinositol 3-kinase	avirulent	Bruckmann A, et al., Microbiology, 2000; 146: 2755.
No	Yes	Yes	PHR1	P43076	pH-responsive protein 1	fungal type cell wall organization, hyphal growth	Glycosidase of cell surface;high pH or filamentation induced	avirulent	De Bernardis F, et al., Infect Immun, 1998; 66: 3317.
No	Yes	Yes	SSN6	Q5ADP3	Transcription regulator	hyphal, filamentous growth	putative global transcriptional co-repressor	avirulent	Hwang C, et al., Mol Microbiol, 2003; 47: 1029.
No	Yes	Yes	SUN41	Q59NP5	Secreted beta-glucosidase SUN41	adhesion, biofilm formation	Putative cell wall glycosidase	avirulent	Norice CT, et al., Eukaryot Cell, 2007; 6: 2046.
No	Yes	Yes	CHS1	Q5A594	Chitin synthase	cell wall biosynthetic process	chitin sythase	avirulent	Munro CA, et al., Mol Microbiol, 2001; 39: 1414.
No	Yes	Yes	CHK1	Q5AHA0	Histidine protein kinase 1	hyphal growth defect	cell wall synthesis	avirulent in mouse	Calera JA, et al., Infect Immun, 1999; 67: 4280.
No	No	Yes	CHO1	A0A1D8PF32	CDP-diacylglycerol--serine O-phosphatidyltransferase	Catalytic activity	phosphatidylethanolamine biosynthesis	avirulent in mouse	Chen YL, et al., Mol Microbiol, 2010; 75:1112.
No	No	Yes	ATP1	A0A1D8PDC4	F1F0 ATP synthase subunit alpha	Produces ATP from ADP	ATP synthesis coupled proton transport	avirulent	Li S, et al., Front Microbiol, 2017; 8: 285.
No	No	Yes	ATP2	A0A1D8PKZ9	F1F0 ATP synthase subunit beta	Produces ATP from ADP	ATP synthesis coupled proton transport	avirulent	Li S, et al., Front Microbiol, 2018; 9: 1025.
No	No	Yes	ADE5,7	A0A1D8PE67	Bifunctional aminoimidazole ribotide synthase/glycinamide ribotide synthase	de novo purine nucleotide biosynthetic pathway	putative aminoimidazole ribotide synthetase (AIRS) and glycinamide ribotide synthetase (GARS)	avirulent	Jezewski S, et al., Microbiology, 2007; 153: 2351.

No	No	Yes	ARP2	AOW31565	Putative component of the Arp2/3 complex	fungal type cell wall organization	Putative component of the Arp2/3 complex	avirulent	Epp E, et al., Mol Microbiol, 2010; 75: 1182.
No	No	Yes	CLA4	Q5APR8	Serine/threonine-protein kinase CLA4	filamentous growth,chlamydospore formation and virulence	activation of protein kinase activity	avirulent	Leberer E, et al., Curr Biol, 1997; 7: 539.
No	No	Yes	GAT1	Q5A432	Transcriptional regulatory protein GAT1	Transcriptional regulator of nitrogen utilization	negative regulation of transcription by RNA polymerase II	avirulent	Limjindaporn T, et al., Mol Microbiol, 2003; 50: 993.
No	No	Yes	HOG1	Q92207	Mitogen-activated protein kinase HOG1	filamentous growth, fungal cell wall organization	mitogen-activated protein (MAP) kinase	avirulent	Cheetham J, et al., J Biol Chem, 2011; 286: 42002.
No	No	Yes	KRE5	A0A1D8PJN6	Kre5p	1,6 beta-glucan biosynthetic process	UDP-glucose:glycoprotein glucosyltransferase (UGGT)	avirulent	Herrero AB, et al., Eukaryot Cell, 2004; 3: 1423.
No	No	Yes	PMR1	A0A1D8PQK6	Calcium-transporting ATPase	cell wall maintenance, protein glycosylation	P-type Ca2+/Mn2+-ATPase	avirulent	Jiang L, et al., Fungal Genet Biol, 2018; 115: 1.
No	No	Yes	SAC1	A0A1D8PEV5	Phosphatidylinositol-3-phosphatase	phosphatidylinositol-4-phosphate phosphatase activity	pathogenesis	avirulent	Zhang B, et al., Fungal Genet Biol, 2015; 81: 261.
No	No	Yes	SSQ1	A0A1D8PRH1	Hsp70 family ATPase	ATPase activity	cellular response to unfolded protein	avirulent	Dong Y, et al., Int J Biochem Cell Biol, 2017; 85: 44.
No	No	Yes	TFP1	Q5AJB1	V-type proton ATPase catalytic subunit A	Catalytic activity	pathogenesis	avirulent	Jia C, et al., Fungal Genet Biol, 2014; 71: 58.
No	No	Yes	VAC1 (PEP7)	Q59UQ8	Pep7p	adhesion, filamentous growth	vesicle transport pt	avirulent	Franke K, et al., Microbiology, 2006; 152: 3111.
No	No	Yes	YPT72	A0A1D8PHR9	Rab family GTPase	GTPase activity	pathogenesis	avirulent	Johnston DA, et al., Infect Immun, 2009; 77: 2343.
No	No	Yes	CMP1	A0A1D8PCA8	Serine/threonine-protein phosphatase	hyphal growth, pH stress	Catalytic subunit of calcineurin	avirulent	Bader T, et al., Infect Immun, 2003; 71: 5344.
No	No	Yes	NAG1	Q04802	Glucosamine-6-phosphate isomerase	N-acetylglucosamine catabolic process	Glucosamine-6-phosphate deaminase	avirulent	Singh P, et al., Infect Immun, 2001; 69: 7898.
No	No	Yes	PMT1	O74189	Dolichyl-phosphate-mannose--protein mannosyltransferase 1	cell wall organization, hyphal growth	isoforms of protein mannosyltransferases	avirulent	Timpel C, et al., J Biol Chem, 1998 ; 273: 20837.

No	No	Yes	VPS4	Q5AG40	Vacuolar protein sorting-associated protein 4	vacuole organization, filamentous growth	Protein involved in transport from multivesicular body (MVB) to the vacuole	avirulent	Lee SA, et al., Mycopathologia, 2009 ; 167: 55.
No	No	Yes	RAD52	Q59NG2	Recombinase	DNA double strand break repair	required for DNA homologous recombination	avirulent	Chauhan N, et al., Infect Immun, 2005 ; 73: 8069.
No	No	Yes	CDC24	AAO25556	GDP-GTP exchange factor for Cdc42p	hyphal growth, pathogenesis	GDP-GTP exchange factor for Cdc42p	avirulent (induc prom)	Bassilana M, et al., Eukaryot Cell, 2003; 2: 9.
No	No	Yes	CEF3 (YEF3/EFT 3)	CAA78282	Translation elongation factor 3	translational elongation	Translation elongation factor 3	avirulent (induc prom)	Nakayama, et al., Infect Immun, 2000; 68: 6712.
No	No	Yes	RHO1	O42825	GTP-binding protein RHO1	1,3 beta-glucan biosynthetic process	GTPase of Rho family>>regulates beta 1,3-glucan synthesis	avirulent (induc.prom)	Smith SE, et al., FEMS Yeast Res, 2002; 2: 103.
No	No	No	ADE2	Q92210	Phosphoribosylaminoimidazole carboxylase	adenine biosynthesis	phosphoribosylaminoimidazole carboxylase	reduced virulence	Kirsch DR, Whitney RR. Infect Immun, 1991; 59: 3297.
No	No	No	AGE3	A0A1D8PCP6	Age3p	pathogenesis	Putative transcription factor with zinc finger DNA-binding motif	reduced virulence	Epp E, et al., PLoS Pathog, 2010; 6: e1000753
No	No	No	ALS1	Q5A8T4	Agglutinin-like protein 1 (Adhesin 1)	cell adhesion, hyphal growth	adhesin	reduced virulence	Fu Y, et al., Mol Microbiol, 2002; 44: 61.
No	No	No	ARF1	P22274	ADP-ribosylation factor	GTP-binding	intracellular protein transport	reduced virulence	Zhang B, et al., Fungal Genet Biol, 2015; 81: 261.
No	No	No	ATC1	Q5AAU5	Cell wall acid trehalase ATC1	trehalose catabolic process	cell wall acid trehalase	reduced virulence	Pedreno Y, et al., FEBS J, 2018; 285: 2004.
No	No	No	ATG8	P0C075	Autophagy-related protein 8			reduced virulence	Li J, et al., Int J Med Microbiol, 2018; 308: 378.
No	No	No	BIG1	Q59WG7	Protein BIG1	adhesion, filamentation	involved in beta-1,6 glucan synthesis	reduced virulence	Umeyama T, et al., Infect Immun, 2006; 74: 2373.
No	No	No	BMH1	O42766	14-3-3 protein homolog	hyphal growth	multifunctional signal-modulating 14-3-3 protein	reduced virulence	Kelly MN, et al., Microbiology, 2009; 155: 1536.

No	No	No	CAS5	Q5AMH6	Cell wall integrity transcriptional regulator CAS5	cell wall damage response	Putative zinc finger transcription factor	reduced virulence	Chamilos G, et al., J Infect Dis, 2009; 200: 152.
No	No	No	CAT1	O13289	Peroxisomal catalase	hydrogen pyroxide catabolic process, hyphal growth	catalase	reduced virulence	Wysong DR, et al., Infect Immun, 1998; 66: 1953.
No	No	No	CCZ1	A0A1D8PQ69	Uncharacterized protein		autophagy	reduced virulence	Dong Y, et al., Int J Biochem Cell Biol, 2015; 69: 41.
No	No	No	CDC10	P39827	Cell division control protein 10	cell cycle	pathogenesis	reduced virulence	Gonzalez-Novo A, et al., Microbiol Immunol, 2006; 50: 499.
No	No	No	CDC11	G1UB61	Cell division control protein 11	cell cycle	pathogenesis	reduced virulence	Warenda AJ, et al., Infect Immun, 2003; 71: 4045.
No	No	No	CEK1	Q5A1D3	Extracellular signal-regulated kinase 1	fungal cell wall biogenesis, hyphal growth	ERK-family protein kinase	reduced virulence	Csank C, et al., Infect Immun, 1998; 66: 2713.
No	No	No	CHS3	A0A1D8PFK5	Chitin synthase	cell wall biosynthetic process	chitin synthase	reduced virulence	Mio T, et al., J Bacteriol, 1996; 178: 2416.
No	No	No	CHS7	Q5AA40	Chitin synthase export chaperone	cell wall biosynthetic process	Protein required for wild-type chitin synthase III activity	reduced virulence	Sanz M,et al., Microbiol, 2005;151: 2623.
No	No	No	CNB1	A0A1D8PP58	Calcineurin regulatory subunit B	hyphal growth, pH stress	Regulatory subunit of calcineurin	reduced virulence	Blankenship JR, et al., Eukaryot Cell, 2003; 2: 422.
No	No	No	CNH1	A0A1D8PKY1	Cnh1p	morphogenesis, metal ion transport	Na+/H+ antiporter	reduced virulence	Soong TW, et al., Microbiology, 2000; 146: 1035.
No	No	No	COS1(NIK1)	Q5A599	Histidine protein kinase NIK1	cell wall biosynthetic process	Histidine kinase	reduced virulence	Selitrennikoff CP, et al., Med Mycol, 2001; 39: 69.
No	No	No	CPH1	P43079	Transcription factor CPH1	mating and hyphal growth	Transcription factor	reduced virulence	Lo JH, et al., Cell, 1997; 90: 939.
No	No	No	CPP1	Q5APU2	Dual specificity protein tyrosine phosphatase CCP1	pheromone response MAP kinase cascade	MAPK phosphatase of the VH1 family	reduced virulence	Csank C, et al., Mol Biol, 1997, Cell; 8: 2539.

No	No	No	CRH11	Q5AFA2	Extracellular glycosidase CRH11	cell wall org	glycosylphosphatidylinositol-dep cell wall pts	reduced virulence	Pardini G, et al., J Biol Chem, 2006; 281: 40399.
No	No	No	CRH12	Q5AK54	Extracellular glycosidase CRH12	cell wall org	glycosylphosphatidylinositol-dep cell wall pts	reduced virulence	Pardini G, et al., J Biol Chem, 2006; 281: 40399.
No	No	No	CSP37	Q5A9D4	Csp37p	pathogenesis, cell wall	Plasma membrane, hyphal cell wall protein	reduced virulence	Sentandreu M, et al., J Bacteriol, 1997; 179: 4654.
No	No	No	CST20	P0CY24	Serine/threonine-protein kinase CST20	protein serine/threonine kinase activity	pathogenesis	reduced virulence	Leberer E, et al., Proc Natl Acad Sci USA, 1996; 93: 13217.
No	No	No	CTF1	A0A1D8PGH7	Ctf1p	fatty acid catabolic process	Putative zinc-finger transcription factor	reduced virulence	Ramirez MA, Lorenz MC. Eukaryot Cell, 2009; 8: 1604.
No	No	No	DPP3	Q5AH74	Bifunctional diacylglycerol diphosphate phosphatase	farnesol biosynthesis	phosphatase, converts farnesyl PP to farnesol	reduced virulence	Navarathna DH, et al., Infect Immun, 2007; 75: 1609.
No	No	No	DRG1	Q5A779	GTP-binding protein	filamentous growth	cytoplasmic G protein>>filamentous growth	reduced virulence	Chen X, Kumamoto CA. Microbiology, 2006; 152: 3691.
No	No	No	ECM25	A0A1D8PFN0	Ecm25p	morphogenesis, cell wall organization	protein involved in cell morphogenesis	reduced virulence	Zhang T, et al., Sci China C Life Sci, 2008; 51: 362.
No	No	No	EFG1	Q59X67	Enhanced filamentous growth protein 1	hyphal growth, metabolism	Transcriptional repressor	reduced virulence	Lo JH, et al., Cell, 1997; 90: 939.
No	No	No	ERG24	AOW27923	sterol C-14 reductase	ergosterol biosynthetic process	sterol C-14 reductase	reduced virulence	Jia N, et al., Antimicrob Agents Chemother, 2002; 46: 947.
No	No	No	ERG3	Q59VG6	C-5 sterol desaturase	ergosterol synthesis, hyphal growth	sterol C5,6-desaturase	reduced virulence	Chau AS, et al., Antimicrob Agents Chemother, 2005; 49: 3646.
No	No	No	FOX2	A0A1D8PJ13	Bifunctional hydroxyacyl-CoA dehydrogenase/enoyl-CoA hydratase	fatty acid B oxidation	peroxisomal enzyme;Predicted 3-hydroxyacyl-CoA epimerase	reduced virulence	Piekarska K, et al., Eukaryot Cell, 2006; 5: 1847.
No	No	No	GLN3	A0A1D8PNX9	Nitrogen-responsive transcriptional regulator	transcriptional regulator	pathogenesis	reduced virulence	Liao WL, et al., Fungal Genet Biol, 2008; 45: 514.
No	No	No	GNA1	Q5AHF9	Glucosamine 6-phosphate N-acetyltransferase	UDP-N-acetylglucosamine biosynthetic process	glucosamine-6-phosphate acetyltransferase	reduced virulence	Mio T, et al., Microbiology, 2000; 146:1753.

No	No	No	GPI7	Q8TGB2	GPI ethanolamine phosphate transferase 2	GPI anchor biosynthesis	Protein involved in attachment of GPI-linked proteins to cell wall	reduced virulence	Richard M, et al., Mol Microbiol, 2002; 44: 841.
No	No	No	GRX2(TTR 1)	Q5ABB1	Dithiol glutaredoxin	pathogenesis, oxidative stress	putative glutathione reductase	reduced virulence	*Chaves* GM, et al., Genet Mol Res, 2007; 6: 1051.
No	No	No	HEM3	O94048	Porphobilinogen deaminase, PBG	heme biosynthetic process	Hydroxymethylbilane synthase (uroporphyrinogen I synthase	reduced virulence	Kirsch DR, Whitney RR. Infect Immun, 1991; 59: 3297.
No	No	No	HGC1	Q5ABE2	Hypha-specific G1 cyclin-related protein 1	hyphal morphogenesis	G1 cyclin-related protein	reduced virulence	Zeng X, et al., EMBO, 2004; 23: 1845.
No	No	No	HGT4	Q5ANE1	Glucose sensor	CHO transport	glucose and galactose sensor	reduced virulence	Brown V, et al., Eukaryot Cell, 2006; 5: 1726.
No	No	No	HPD1	A0A1D8PQ07	Hpd1p	3-hydroxyisobutyrate dehydrogenase activity	propionyl-CoA catabolic process	reduced virulence	Otzen C, et al., J Biol Chem, 2014; 289: 8151.
No	No	No	HSL1	Q5AG71	Serine/threonine-protein kinase HSL1	filamentous growth	Ser/Thr protein kinase	reduced virulence	Umeyama T, et al., Mol Microbiol, 2005; 55: 381.
No	No	No	HWP1	P46593	Hyphal wall protein 1(Cell elongation protein 2)	adhesion,cell wall organization	hyphal cell wall protein	reduced virulence	Tsuchimori N, et al., Infect Immun, 2000; 68: 1997.
No	No	No	HWP2	Q59PF9	Hyphal wall protein 2(GPI-anchored protein 8)	filamentous growth, pathogenesis	GPI-anchored protein	reduced virulence	Hayek P, et al., Microbiol Res, 2010; 165: 250.
No	No	No	IFF11	Q5A7R7	Cell wall protein IFF11 (Adhesin-like protein IFF11)	cell wall structure, virulence	Secreted protein	reduced virulence	Bates S, et al., Infect Immun, 2007; 75: 2922.
No	No	No	IFF4	Q5AAL9	Cell wall protein IFF4(Adhesin-like protein IFF4)	adhesion, pathogenesis	GPI anchor protein	reduced virulence	Kempf M, et al., Mycopathologia, 2009; 168: 73.
No	No	No	INP51	A0A1D8PIH6	Phosphoinositide 5-phosphatase	cell integrity pathway, hyphal growth	phosphatidylinositol-(4,5)-bisphosphate [PI(4,5)P2] 5-phosphatase	reduced virulence	Badrane H, et al., Microbiology, 2008; 154: 3296.
No	No	No	INT1	P53705	Bud site selection protein BUD4	morphogenesis, adhesion	Integrin-like protein	reduced virulence	Gale, CA, et al., Science, 1998; 279: p1355.
No	No	No	IRS4	Q59SR6	Increased rDNA silencing protein 4	adhesion, cell wall organization	Protein with roles in cell wall integrity	reduced virulence	Badrane H, et al., Microbiology, 2005; 151: 2923.

No	No	No	KEX2	A0A1D8PEG3	Kexin	hyphal growth, pathogenesis	Subtilisin-like protease (proprotein convertase)	reduced virulence	Newport G, et al., J.Biol.Chem. 2003; 278:1713.
No	No	No	LIG4	P52496	DNA ligase 4	DNA double strand break repair	DNA ligase	reduced virulence	Andaluz E, et al., Infect Immun, 2001; 69: 137.
No	No	No	MAD2	Q59VQ3	Spindle assembly checkpoint component MAD2	mitotic cell cycle spindle assembly	spindle assembly checkpoint (SAC) component	reduced virulence	Bai C, et al., Mol Microbiol, 2002; 45: 31.
No	No	No	MCA1	Q5ANA8	Metacaspase-1	apoptosis	apoptotic process	reduced virulence	Jeong J, et al., Fungal Genet Biol, 2016; 97: 18.
No	No	No	MDR1	Q5ABU7	Multidrug resistance protein 1 (Benomyl resistance protein 1)	biofilm formation, drug export	Multidrug efflux pump of plasma membrane	reduced virulence	Becker JM, et al., Infect Immun, 1995; 63: 4515.
No	No	No	MDS3	Q59WF4	Negative regulator of sporulation MDS3	pH induced hyphal formation	required for growth and hyphal formation at alkaline pH	reduced virulence	Davis DA, et al., Genetics, 2002; 162: 1573.
No	No	No	MIT1	A0A1D8PIF7	Mannosylinositol phosphorylceramide synthase catalytic subunit	glycosphingolipid synthesis	GDP-mannose:inositol-phospho-ceramide mannose transferase	reduced virulence	Mille C, et al., J Biol Chem, 2004; 279: 47952.
No	No	No	MKC1	Q5AAG6	Mitogen-activated protein kinase MKC1	filamentous, hyphal growth	mitogen-activated protein kinase	reduced virulence	Diez-Orejas R, et al., Infect Immun, 1997; 65: 833.
No	No	No	MLT1	Q5A762	Multiple drug resistance-associated protein-like transporter 1	Vacuolar multi-drug resistance ABC transporter	pathogenesis	reduced virulence	Khandelwal NK, et al., Biochem J, 2016; 473: 1537.
No	No	No	MNN5 (MNN2)	Q59WF4	Alpha-1,2-mannosyltransferase MNN2	filamentous, hyphal growth	alpha-1,2-mannosyltransferase	reduced virulence	Bai C, et al., Eukaryot Cell, 2006; 5: 238.
No	No	No	MNT1	Q00310	Glycolipid 2-alpha-mannosyltransferase 1	adhesion, filamentous growth	Alpha-1,2-mannosyl transferase	reduced virulence	Buurman ET, et al., Proc Natl Acad Sci U S A, 1998; 95: 7670.
No	No	No	MRS4	Q5A2T7	Fe(2+) transporter	iron ion transmembrane transporter activity	iron ion homeostasis	reduced virulence	Xu N, et al., Biochim Biophys Acta, 2014; 1843: 629.
No	No	No	NAG2 (DAC1)	A0A1D8PQG3	N-acetylglucosamine-6-phosphate deacetylase	N-acetylglucosamine catabolic process	N-acetylglucosamine-6-phosphate (GlcNAcP) deacetylase	reduced virulence	Yamada-Okabe T, et al., Eur J Biochem, 2001; 268: 2498.
No	No	No	NAG3	A0A1D8PQG0	Major facilitator superfamily multidrug transporter NAG3	drug transport	Putative transporter of the major facilitator superfamily (MFS)	reduced virulence	Yamada-Okabe T, Yamada-Okabe H. FEMS Microbiol Lett, 2002; 212: 15.

No	No	No	NAG4	Q59RG0	Major facilitator superfamily multidrug transporter NAG4	drug transport	Putative transporter	reduced virulence	Yamada-Okabe T, Yamada-Okabe H. FEMS Microbiol Lett, 2002; 212: 15.
No	No	No	NAG6	Q9C0L9	Protein SEY1	hexose transport	Protein required for wild-type mouse virulence and wild-type cycloheximide resistance	reduced virulence	Yamada-Okabe T, Yamada-Okabe H. FEMS Microbiol Lett, 2002; 212: 15.
No	No	No	NOT5	A0A1D8PE87	CCR4-NOT core subunit	adhesion, hyphal growth	member of CCR-NOT complex>>global regulator of transcription	reduced virulence	Cheng S, et al., Infect Immun, 2005; 73: 7190.
No	No	No	NRG1	Q5A0E5	Transcriptional regulator NRG1	hyphal growth	Transcriptional repressor	reduced virulence	Murad AM, et al., EMBO J, 2001; 20: 4742.
No	No	No	PDE2	A0A1D8PCV9	Phosphodiesterase	cAMP-mediated signal transduction pathway for morphologic switching (hyphal formation)	phosphodiesterase>>high affinity for cAMP	reduced virulence	Bahn YS, et al., Mol Microbiol, 2003; 50: 391.
No	No	No	PEP12	A0A1D8PNI0	SNAP receptor	vacuolar transport, endocytosis, and secretion	t-SNARE (pre-vacuolar trafficking gene)	reduced virulence	Palanisamy SK, et al., Eukaryot Cell, 2010; 9: 266.
No	No	No	PGA13	Q5A343	GPI-anchored protein 13	cell wall synthesis	pathogenesis	reduced virulence	Gelis S, et al., Fungal Genet Biol, 2012; 49: 322.
No	No	No	PHO4	A0A1D8PMD1	Phosphate-sensing transcription factor	protein dimerization activity	cellular phosphate ion homeostasis	reduced virulence	Ikeh MA, et al., Mol Biol Cell, 2016; 27: 2784.
No	No	No	PLB1	Q9UWF6	Lysophospholipase 1	migration in host, pathogenesis	phospholipase B	reduced virulence	Leidich SD, et al., J Biol Chem, 1998; 273: 26078.
No	No	No	PLB5	A0A1D8PEB1	Lysophospholipase	multigene phospholipase family	phospholipase B	reduced virulence	Theiss S, et al., Int J Med Microbiol, 2006; 296: 405.
No	No	No	PLD1	A0A1D8PF62	Phospholipase	hyphal growth, pathogenesis	Phospholipase D1	reduced virulence	Dolan JW, et al., Med Mycol, 2004; 42: 439.
No	No	No	PMT4	Q59X23	Dolichyl-phosphate-mannose--protein mannosyltransferase 4	cell wall organization, hyphal growth	isoforms of protein mannosyltransferases	reduced virulence	Rouabhia M, et al., Infect Immun, 2005; 73: 4571.

No	No	No	PMT5	Q5ACU3	Dolichyl-phosphate-mannose--protein mannosyltransferase 5	cell wall organization, hyphal growth	isoforms of protein mannosyltransferases	reduced virulence	Rouabhia M, et al., Infect Immun, 2005; 73: 4571.
No	No	No	PMT6	Q5A688	Dolichyl-phosphate-mannose--protein mannosyltransferase 6	cell wall organization, hyphal growth	Protein mannosyltransferases	reduced virulence	Timpel C, et al., J Bacteriol, 2000 ; 182: 3063.
No	No	No	RAS1	Q59XU5	Ras-like protein 1 (Ras homolog type B)	cAMP-mediated signaling	RAS signal transduction GTPase	reduced virulence	Leberer E, et al., Mol Microbiol, 2001; 42: 673.
No	No	No	Rvs161	Q5AFE4	Regulator of cytoskeleton and endocytosis RVS161	cytoskeletal protein binding	pathogenesis	reduced virulence	Douglas LM, et al., Infect Immun, 2009; 77: 4150.
No	No	No	Rvs167	Q59LF3	Regulator of cytoskeleton and endocytosis RVS167	cytoskeletal protein binding	pathogenesis	reduced virulence	Douglas LM, et al., Infect Immun, 2009; 77: 4150.
No	No	No	RCH1	Q59UQ7	Solute carrier RCH1	negatively regulates the cytosolic homeostasis	calcium ion import	reduced virulence	Jiang L, et al., Fungal Genet Biol, 2018; 115: 1.
No	No	No	RFX2	Q5AMQ6	RFX-like DNA-binding protein RFX2	filamentation, adhesion	Transcriptional repressor	reduced virulence	Hao B, et al., Eukaryot Cell, 2009; 8: 627.
No	No	No	RIM101	Q9UW14	pH-response transcription factor pacC	filamentous, hyphal growth	Transcription factor involved in alkaline pH response	reduced virulence	Davis D, et al., Infect Immun, 2000; 68: 5953.
No	No	No	RIM8	Q9UW13	pH-response regulator protein palF	filamentous, hyphal growth	Transcription factor involved in alkaline pH response	reduced virulence	Davis D, et al., Infect Immun, 2000; 68: 5953.
No	No	No	RVS161	Q5AFE4	Regulator of cytoskeleton and endocytosis RVS161	endocytosis, cell wall organization	Amphiphysin membrane protein containing a BAR domain	reduced virulence	Douglas LM, et al., Infect Immun, 2009; 77: 4150.
No	No	No	RVS167	Q59LF3	Regulator of cytoskeleton and endocytosis RVS167	endocytosis, cell wall organization	Protein containing a BAR domain	reduced virulence	Douglas LM, et al., Infect Immun, 2009; 77: 4150.
No	No	No	SAP7	Q59VH7	Candidapepsin-7	protein metabolic process, pathogenesis	Member of the secreted aspartyl proteinase family	reduced virulence	Taylor BN, et al., Infect Immun, 2005; 73: 7061.
No	No	No	SDH2	Q59QN7	Succinate dehydrogenase [ubiquinone] iron-sulfur subunit	transferring electrons from succinate to ubiquinone	respiratory electron transport chain	reduced virulence	Bi S, et al., Future Microbiol, 2018; 13: 1141.

No	No	No	SEF1	Q59UY7	Transcriptional regulatory protein SEF1	Transcription factor	pathogenesis	reduced virulence	Chen C, et al., Cell Host Microbe, 2011; 10: 118.
No	No	No	SET1	Q5ABG1	Histone-lysine N-methyltransferasedomain-containing protein 1)	cellular developmental process, filamentation	putative lysine histone methyltransferase	reduced virulence	Raman SB, et al., Mol Microbiol, 2006; 60: 697.
No	No	No	SFL1	Q5A287	Transcription factor SFL1	filamentous growth, flocculation	transcriptional repressor	reduced virulence	Li Y, et al., Eukaryot Cell, 2007; 6: 2112.
No	No	No	SHO1	Q5AQ36	High osmolarity signaling protein SHO1	osmatic & oxidative stress	Predicted adaptor protein involved in activation of MAP kinase-dependent signaling pathways	reduced virulence	Roman E, et al., Eukaryot Cell, 2009; 8: 1235.
No	No	No	SIT4	Q59KY8	Serine/threonine-protein phosphatase SIT4	hyphal growth, pathogenesis	catalytic subunit of a type2A-related protein phosphatase	reduced virulence	Lee CM, et al., Mol Microbiol, 2004; 51: 691.
No	No	No	SKN7	Q5A4X5	Transcription factor SKN7	response to oxidative stress, filamentous growth	response regulator protein in a phosphorelay signal transduction pathway	reduced virulence	Singh P, et al., Infect Immun, 2004; 72: 2390.
No	No	No	SLK19	Q5ADT0	Slk19p	cell wall organization	alkaline-induced membrane pt	reduced virulence	Galan A, et al., Microbiology, 2004; 150: 2641.
No	No	No	SLR1	A0A1D8PIP0	Slr1p	RNA binding	pathogenesis	reduced virulence	Ariyachet C, et al., Infect Immun, 2013; 81: 1267.
No	No	No	SOD1	A0A1D8PLJ3	Superoxide dismutase	hyphal growth, response to oxidative stress	Cytosolic copper- and zinc-containing superoxide dismutase	reduced virulence	Hwang CS, et al., Microbiology, 2002; 148: 3705.
No	No	No	SOD5	Q5AD07	Cell surface Cu-only superoxide dismutase 5	pathogenesis, response to oxidative stress	Copper- and zinc-containing superoxide dismutase	reduced virulence	Martchenko M, et al., Mol Biol Cell, 2004; 15: 456.
No	No	No	SUT1	A0A1D8PNM7	Sut1p	DNA-binding transcription factor activity	pathogenesis	reduced virulence	Xu W, et al., PLoS Biol, 2015; 13: e1002076.
No	No	No	SWE1	Q5AP97	Mitosis inhibitor protein kinase SWE1	morphogenesis, pathogenesis	morphogenesis checkpoint kinase	reduced virulence	Gale CA, et al., Microbiol, 2009; 155: 3847.
No	No	No	TEC1	Q5ANJ4	Transcription activator TEC1	cell adhesion, filamentous growth	TEA/ATTS transcription factor	reduced virulence	Schweizer A, et al., Mol Microbiol, 2000; 38: 435.
No	No	No	TPK2	A0A1D8PHU1	cAMP-dependent protein kinase catalytic subunit	morphogenesis, hyphal growth	catalytic subunit of a protein kinase A (PKA) isoform	reduced virulence	Sonneborn A, et al., Mol Microbiol, 2000; 35: 386.

No	No	No	TPS2	Q5AI14	Trehalose-phosphatase	stress response, hyphal growth	trehalose-6-phospate phosphatase	reduced virulence	Zaragoza O, et al., Microbiology, 2002; 148: 1281.
No	No	No	UME6	Q59MD2	Transcriptional regulatory protein UME6	hyphal growth, pathogenesis	Transcription factor	reduced virulence	Banerjee M, et al., Mol Biol Cell, 2008; 19: 1354.
No	No	No	URA3	P13649	Orotidine 5'-phosphate decarboxylase	adhesion, filamentous growth	orotidine 5'-monophosphate decarboxylase	reduced virulence	Kirsch DR, Whitney RR. Infect Immun, 1991; 59: 3297.
No	No	No	UTR2	Q5AJC0	Extracellular glycosidase UTR2	cell wall organization, hyphal growth	GPI anchored cell wall putative glycosidase	reduced virulence	Pardini G, et al., J Biol Chem, 2006; 281: 40399.
No	No	No	VPH2	A0A1D8PFF4	Vph2p	vacuolar proton-transporting V-type ATPase complex assembly		reduced virulence	Jia C, et al., Fungal Genet Biol, 2018; 114: 1.
No	No	No	VPS21	Q59X89	Rab family GTPase	protein transport to vacuole	Rab GTPase	reduced virulence	Johnston DA, et al., Infect Immun, 2009; 77: 2343.
No	No	No	VPS28	Q59SD1	Vacuolar protein sorting-associated protein 28	cell wall organization, pH response	Protein involved in proteolytic activation of Rim101p	reduced virulence	Cornet M, et al., Infect Immun, 2005; 73: 7977.
No	No	No	VPS32	Q5ABD0	Vacuolar-sorting protein SNF7	filamentous growth, cell wall organization	Protein involved in proteolytic activation of Rim101p	reduced virulence	Cornet M, et al., Infect Immun, 2005; 73: 7977.
No	No	No	YHB1	Q59MV9	Flavohemoprotein	nitric oxide dioxygenase activity	pathogenesis	reduced virulence	Hromatka BS, et al., Mol Biol Cell, 2005; 16: 4814.
No	No	No	YPT72	A0A1D8PHR9	Rab family GTPase	GTPase activity	pathogenesis	reduced virulence	Johnston DA, et al., Infect Immun, 2009; 77: 2343.
No	No	No	YSY6	A0A1D8PJJ9	Stress-associated endoplasmic reticulum protein		protein transport	reduced virulence	Li J, et al., Int J Med Microbiol, 2018; 308: 378.
No	No	No	YVC1	Q5A2J7	Calcium channel YVC1	hyphal growth, pathogenesis	protein phosphatase	reduced virulence	Yu Q, et al., Int J Med Microbiol, 2014; 304: 339.
No	No	No	YVH1	A0A1D8PSH7	Tyrosine protein phosphatase	protein tyrosine/serine/threonine phosphatase activity	pathogenesis	reduced virulence	Hanaoka N, et al., Microbiology, 2005; 151: 2223.

No	No	No	PMT2	Q5ADM9	Dolichyl-phosphate-mannose--protein mannosyltransferase 2	cell wall organization, hyphal growth	isoforms of protein mannosyltransferases	reduced virulence (heterozygote)	Rouabhia M, et al., Infect Immun, 2005; 73: 4571.
No	No	No	FBA1	Q9URB4	Fructose-bisphosphate aldolase	glycolysis	fructose-1,6-bisphophate aldolase	reduced virulence (induc prom)	Rodaki A, et al., Eukaryot Cell, 2006; 5: 1371.
No	No	No	NMT1	P30418	Glycylpeptide N-tetradecanoyltransferase	drug binding, pathogenesis	MyristoylCoA:protein N-myristoyltransferase	reduced virulence (knock-down strain)	Weinberg RA, et al., Mol Microbiol, 1995; 16: 241.
No	No	No	SAP4	Q5A8N2	Candidapepsin-4	adhesion, pathogenesis	secreted aspartyl proteinases	reduced virulence of triple deletion mutant	Sanglard D, et al., Infect Immun, 1997; 65: 3539.
No	No	No	SAP5	P43094	Candidapepsin-5	adhesion, pathogenesis	secreted aspartyl proteinases	reduced virulence of triple deletion mutant	Sanglard D, et al., Infect Immun, 1997; 65: 3539.
No	No	No	SAP6	Q5AC08	Candidapepsin-6	adhesion, pathogenesis	secreted aspartyl proteinases	reduced virulence of triple deletion mutant	Sanglard D, et al., Infect Immun, 1997; 65: 3539.
No	No	No	ATP18	A0A1D8PG50	F1F0 ATP synthase subunit i/j	proton transmembrane transporter activity	ATP synthesis coupled proton transport	reduced virulence	Zhao Y, et al., Med Mycol, 2021; 59: 639.

Supplementary Table 2. Summary of F_1F_o-ATP synthase subunits of *C. albicans.*

C. albicans gene name	Protein accession	Protein name	Function	Biological process	Status
ATP1	A0A1D8PDC4	F_1F_o-ATP synthase subunit α	produces ATP from ADP	ATP synthesis coupled proton transport	Protein inferred from homology
ATP2	A0A1D8PKZ9	F_1F_o-ATP synthase subunit β	produces ATP from ADP	ATP synthesis coupled proton transport	Protein inferred from homology
ATP3	A0A1D8PRY3	F_1F_o-ATP synthase subunit γ	produces ATP from ADP	ATP synthesis coupled proton transport	Protein inferred from homology
ATP4	Q59ZE0	F_1F_o-ATP synthase subunit 4	produces ATP from ADP	ATP synthesis coupled proton transport	Protein predicted
ATP5	Q5A7P7	F_1F_o-ATP synthase subunit 5	produces ATP from ADP	ATP synthesis coupled proton transport	Protein inferred from homology
ATP6	Q9B8D4 (I2BJX7)	F_1F_o-ATP synthase subunit a	produces ATP from ADP	ATP synthesis coupled proton transport	Protein inferred from homology
ATP7	Q59PV8	F_1F_o-ATP synthase subunit d	produces ATP from ADP	ATP synthesis coupled proton transport	Protein inferred from homology
ATP8	Q9B8D3	F_1F_o-ATP synthase subunit 8 (A6L)	produces ATP from ADP	ATP synthesis coupled proton transport	Protein inferred from homology
ATP9	Q9B8D5	F_1F_o-ATP synthase subunit 9	produces ATP from ADP	ATP synthesis coupled proton transport	Protein inferred from homology
ATP14	A0A1D8PHL7	F_1F_o-ATP synthase subunit h	produces ATP from ADP	ATP synthesis coupled proton transport	Protein predicted
orf19.5597.1	A0A1D8PQ38	predicted F_1F_o–ATP synthase subunit ε	produces ATP from ADP	ATP synthesis coupled proton transport	Protein inferred from homology
ATP16	A0A1D8PUD2	F_1F_o-ATP synthase subunit δ	produces ATP from ADP	ATP synthesis coupled proton transport	Protein inferred from homology
ATP17	A0A1D8PRM5	F_1F_o-ATP synthase subunit f	produces ATP from ADP	ATP synthesis coupled proton transport	Protein predicted
ATP18	A0A1D8PG50	F_1F_o-ATP synthase subunit i/j	produces ATP from ADP	ATP synthesis coupled proton transport	Protein predicted
ATP19	A0A1D8PFD4	F_1F_o-ATP synthase subunit k	produces ATP from ADP	ATP synthesis coupled proton transport	Protein predicted
ATP20	Q59M30	F_1F_o-ATP synthase subunit g	produces ATP from ADP	ATP synthesis coupled proton transport	Protein inferred from homology
TIM11	A0A1D8PL02	F_1F_o-ATP synthase subunit e	Produces ATP from ADP	ATP synthesis coupled proton transport	Protein inferred from homology

Supplementary Table 3. Strains used in this study.

Strain	Parent	Genotype	Reference
SC5314		Wild-type strain	1
ATP16/*atp16*Δ	SC5314	orf19.7678-1Δ::FRT/orf19.7678-2	
*atp16*Δ/Δ	*ATP16*/*atp16*Δ	orf19.7678-1Δ::FRT/orf19.7678-2Δ::FRT	This study
*atp16*Δ/*ATP16*	*atp16*Δ/Δ	orf19.7678-1Δ::FRT/orf19.7678-2::FRT	This study
Atp16 O/E	SC5314	*ADH1/adh1*::P*ADH1*-orf19.7678-1-*caSAT1*	This study
SN250		wild-type (leu2Δ::C.d.HIS1/leu2Δ::C.m.LEU2, ura3Δ/URA3, his1Δ/his1Δ, arg4Δ/arg4Δ, iro1Δ/IRO1, MTLa/ MTLα)	2
*ras1*Δ/Δ		ura3::limm434/ura3::limm434 ras1::hisG/ras1::hisG:URA3	3

Supplementary Table 4. Primers used in this study.

Primers	Sequence (5’ to 3’) [a]
*atp16*Δ/Δ and *atp16*Δ/*ATP16* construction	
ATP16-1	CCCgggcccATCAACGACATGCAGAAC
ATP16-2	CCCctcgagGCCTCGGTGGCATAAGTT
ATP16-3	ATAAGAATgcggccgcAGAGCTGAATCTAGTGATG
ATP16-4	TCCccgcggTAAGCACTAAAGAAGGGT
CassetF	GCTTTCGGTCGCTGTTCTCA
CassetR	TGTTAGGCGTCATCCTGTGC
ATP16-5	AAGTCATAGAAGMAGCGGCAAG
ATP16-6	TCCAGACAGTCGAGTTAG
ATP16-7	TCTCGGGAGCACAGGATG
ATP16-8	GTTGACTGGTGGCGTAGC
ATP16-F	GAAATTATCCTTGGCATTG
ATP16-R	TGGTCAGAGTCTCCGTTT
Atp16 O/E construction	
ATP16orf-F	CCCctcgagATGTTCAGACAAGTTTTCCGTCAAG
ATP16orf-R	CCCagatctTTACTTAGTAAAATGTTGTAAAGC
pADH1E2-ATP16-up-F	TCTTGTTCGAAACGGCAGTGCCTC
pADH1E2-ATP16-up-R	AACCAGATTTCCAGATTTCCAG
pADH1E2-ATP16-down-F	TAAGCAGACAGCTCCTTGGCATAC
pADH1E2-ATP16-down-R	GAGTTGTTGGAGAGGTAAACCCAG
RT-qPCR	
ATP16-RTF	AACTTATGCCACCGAGGC
ATP16-RTR	CATCACTAGATTCAGCTCT
SSA1-F	ATTGCTGAAGGTTATTTGGGTTC
SSA1-R	GGTGGCTTGTCTTTGAGAATC
HWP1-F	TGTCTACACTACATTCTGTC
HWP1-R	AGGAATAGATGGTTGTGAAC
ALS3-F	CTCATTACACCAACCATACA
ALS3-R	GGATTCTGTGGTTGTAGTAT
HGC1-F	GTATCGCTGGTTCTCGTGCT
HGC1-R	AGGTGTACCACTACCACCATT
RAS1-F	ATCAAGATGGATTAGCATTGG
RAS1-R	TGTTGTTGCTGTTGTTGTTG
CYR1-F	AGAAAGAAGACGATGAAACAG
CYR1-R	AGGAGAACTAGAGGATGTAGAC
TPK1-F	AGAACTTGCCAACAAACAAC
TPK1-R	TTTCTTGGTCAAGGAAAGAC
TPK2-F	TTGTTGCCTGAACGTTCTAC
TPK2-R	CTACCATTGTGAACTGATCTC

EFG1-F	ACAACCTCAGCATTACAATG
EFG1-R	ATAGGTACTGCTTGTTGACC
FLO8-F	AGCAAATGACTAAGATGGCTG
FLO8-R	AGTCGGAATTACCAGTGTTTC
CPH1-F	ATGCAACACTATTTATACCTC
CPH1-R	CGGATATTGTTGATGATGATA
TEC1-F	AGGTTCCCTGGTTTAAGTG
TEC1-R	ACTGGTATGTGTGGGTGAT
COX1-F	GCACTTCTTAGGATTGAATG
COX1-R	TTCTTCACGCTCATTCTCAC
COX2-F	AGATGCTACTCCAGGTCG
COX2-R	ACTGCTCTACCATTGAGC
COX3-F	CCCTACTATATCACCAGCTG
COX3-R	TCACTAAGTGTAAATCCAGC
COB-F	GATAGATTGCCAATGCATCC
COB-R	CGAAGTATAGCATAGAATGG
ATP6-F	GCTGGAGGTCATTTCTTCCCT
ATP6-R	TAGCACCAAGACGTAGACCT
ATP8-F	CAGGTATTGCAGCTGTATCAA
ATP8-R	AGCTCTAGCTACTAATAAACGTA
ATP9-F	ACAGGTATTGCAGCTGTATCAAT
ATP9-R	AGCTCTAGCTACTAATAAACGTAAT

[a] Lowercase sequences represent the following restriction sites: ApaI for ATP16-1, XhoI for ATP16-2, NotI for ATP16-3, SacII for ATP16-4.

Supplementary Table 5. The mean fluorescence intensity value of ROS.

Strains	P1 Mean Fluorescence Intensity (PE)		
	1st experiment	2nd experiment	3rd experiment
WT	2181	1205	1361
*atp16*Δ/Δ	1795	1197	1243
*atp16*Δ/*ATP16*	2635	1116	1352

Supplementary Table 6. The red/green mean fluorescent intensity value of ΔΨm.

Strains	P1 Mean Fluorescence Intensity (PE)					
	1st experiment		2nd experiment		3rd experiment	
	JC-1 Red	JC-1 green	JC-1 Red	JC-1 green	JC-1 Red	JC-1 green
WT	6544	8971	23322	31970	11599	17164
*atp16*Δ/Δ	2954	7766	6310	16929	15127	46298
*atp16*Δ/*ATP16*	4351	6800	13057	15592	17026	22645

Supplementary References

1. Gillum, A. M., Tsay, E. Y. & Kirsch, D. R. Isolation of the *Candida albicans* gene for orotidine-5'-phosphate decarboxylase by complementation of *S. cerevisiae ura3* and *E. coli pyrF* mutations. *Mol. Gen. Genet.* **198**, 179-182 (1984).
2. Noble, S. M., French, S., Kohn, L. A., Chen, V. & Johnson, A. D. Systematic screens of a *Candida albicans* homozygous deletion library decouple morphogenetic switching and pathogenicity. *Nat. Genet.* **42**, 590-598 (2010).
3. Grahl, N., *et al.* Mitochondrial Activity and Cyr1 Are Key Regulators of Ras1 Activation of *C. albicans* Virulence Pathways. *PLoS pathog.* **11**, e1005133 (2015).
