# Supplementary material for: The δ subunit of F1Fo-ATP synthase is required for pathogenicity of Candida albicans
Source: Nat Commun. 2021 Oct 15;12:6041. doi: 10.1038/s41467-021-26313-9 (PMC8519961; doi:10.1038/s41467-021-26313-9)
Supplement: Supplementary file 2 — Reporting Summary [file 41467_2021_26313_MOESM2_ESM.pdf]

nature research

Corresponding author(s): Hong Zhang

Last updated by author(s): Sep 22, 2021

Reporting Summary

Nature Research wishes to improve the reproducibility of the work that we publish. This form provides structure for consistency and transparency in reporting. For further information on Nature Research policies, see our Editorial Policies and the Editorial Policy Checklist.

Statistics

For all statistical analyses, confirm that the following items are present in the figure legend, table legend, main text, or Methods section.

n/a	Confirmed	
☐	☒	The exact sample size (*n*) for each experimental group/condition, given as a discrete number and unit of measurement
☐	☒	A statement on whether measurements were taken from distinct samples or whether the same sample was measured repeatedly
☐	☒	The statistical test(s) used AND whether they are one- or two-sided<br>*Only common tests should be described solely by name; describe more complex techniques in the Methods section.*
☒	☐	A description of all covariates tested
☒	☐	A description of any assumptions or corrections, such as tests of normality and adjustment for multiple comparisons
☐	☒	A full description of the statistical parameters including central tendency (e.g. means) or other basic estimates (e.g. regression coefficient) AND variation (e.g. standard deviation) or associated estimates of uncertainty (e.g. confidence intervals)
☐	☒	For null hypothesis testing, the test statistic (e.g. *F*, *t*, *r*) with confidence intervals, effect sizes, degrees of freedom and *P* value noted<br>*Give P values as exact values whenever suitable.*
☒	☐	For Bayesian analysis, information on the choice of priors and Markov chain Monte Carlo settings
☒	☐	For hierarchical and complex designs, identification of the appropriate level for tests and full reporting of outcomes
☒	☐	Estimates of effect sizes (e.g. Cohen's *d*, Pearson's *r*), indicating how they were calculated

Our web collection on statistics for biologists contains articles on many of the points above.

Software and code

Policy information about availability of computer code

Data collection	Bio-Rad CFX manager 3.1 for Bio-Rad CFX96 Real-Time system. Inveon Research Workspace 4.2 for Inveon PET/CT scanner. Wave 2.6 for Seahorse Xfe96 analyser. BD FACSDiva v8.0.1 for BD FACScan flow cytometer. ZEN 2.3 for Carl Zeiss confocal laser scanning microscope. MOE v2015.1001 software for homology modelling and virtual screening. ImageJ for Western blots.
Data analysis	Graphpad Prism 8.0.1

For manuscripts utilizing custom algorithms or software that are central to the research but not yet described in published literature, software must be made available to editors and reviewers. We strongly encourage code deposition in a community repository (e.g. GitHub). See the Nature Research guidelines for submitting code & software for further information.

Data

Policy information about availability of data

All manuscripts must include a data availability statement. This statement should provide the following information, where applicable:

- Accession codes, unique identifiers, or web links for publicly available datasets
- A list of figures that have associated raw data
- A description of any restrictions on data availability

Proteomics data are deposited in the Pride database under the accession code PXD024729. Metabolomics data are deposited in the MetaboLights database under the accession code MTBLS3332. All raw data used to generate main manuscript and supplementary figures can be found in the Source Data file provided with this paper.

Field-specific reporting

Please select the one below that is the best fit for your research. If you are not sure, read the appropriate sections before making your selection.

☒ Life sciences ☐ Behavioural & social sciences ☐ Ecological, evolutionary & environmental sciences

For a reference copy of the document with all sections, see nature.com/documents/nr-reporting-summary-flat.pdf

Life sciences study design

All studies must disclose on these points even when the disclosure is negative.

Sample size	Sample size was always n=3 or greater when statistical analysis was required. Sample size of animal was determined based on the published literature (PMID 15962230). All experiments were performed in biological duplicate or greater with little deviation between replicates.
Data exclusions	No data were excluded from the analyses.
Replication	All attempts at replication were successful. The number of replicates is indicated in the corresponding figure legend and/or in the corresponding material and method section.
Randomization	Animal were randomized during group allocation. Other samples were randomly distributed into experimental group.
Blinding	Animal studies were blinded during group allocation and experimentation. Other experiments were also blinded during group allocation and experimentation. These assays had a quantitative output, and therefore, blinding was not required during analysis to eliminate user bias.

Reporting for specific materials, systems and methods

We require information from authors about some types of materials, experimental systems and methods used in many studies. Here, indicate whether each material, system or method listed is relevant to your study. If you are not sure if a list item applies to your research, read the appropriate section before selecting a response.

Materials & experimental systems

n/a	Involved in the study	
☐	☒	Antibodies
☐	☒	Eukaryotic cell lines
☒	☐	Palaeontology and archaeology
☐	☒	Animals and other organisms
☒	☐	Human research participants
☒	☐	Clinical data
☒	☐	Dual use research of concern

Methods

n/a	Involved in the study	
☒	☐	ChIP-seq
☐	☒	Flow cytometry
☒	☐	MRI-based neuroimaging

Antibodies

Antibodies used	monoclonal anti-Ras clone 10 (1:1000; Millipore # 05-516), goat anti-mouse (1:10000; EarthOx Life Sciences # E030110), GAPDH polyclonal antibody (1:5000; Bioworld # AP0063), anti-phosphorylated tyrosine (p-Tyr) antibody (1:1000; PTM BIO # PTM-701), anti-phosphorylated threonine (p-Thr) antibody (1:100; Abcam # ab9337) and anti-phosphorylated serine (p-Ser) antibody (1:800; Abcam # ab6639).
Validation	All the antibodies used in the study were bought commercially, and have been validated for intended uses by the manufacturer as stated on their websites. Manufacturer citations are listed in manufacturer websites for each specific antibody.<br>monoclonal anti-Ras clone 10 (1:1000; Millipore # 05-516) (https://www.merckmillipore.com/HK/en/product/Anti-Ras-Antibody-clone-RAS10,MM_NF-05-516), goat anti-mouse (1:10000; EarthOx Life Sciences # E030110) (https://earthox.net/?product=hrp-goat-anti-mouse-igghl-e030110-02), GAPDH polyclonal antibody (1:5000; Bioworld # AP0063) (https://www.citeab.com/antibodies/2206645-ap0063-gapdh-polyclonal-antibody), anti-phosphorylated tyrosine (p-Tyr) antibody (1:1000; PTM BIO # PTM-701) (https://www.ptmbiolabs.com/product/ptm-701/), anti-phosphorylated threonine (p-Thr) antibody (1:100; Abcam # ab9337) (https://www.abcam.com/phosphothreonine-antibody-ab9337.html) and anti-phosphorylated serine (p-Ser) antibody (1:800; Abcam # ab6639) (https://www.abcam.com/phosphoserine-antibody-psr-45-ab6639.html?productWallTab=Abreviews).

Eukaryotic cell lines

Policy information about cell lines

Cell line source(s)	RAW264.7 macrophage cell was sourced from ATCC TIB-71.

Authentication	The purchased cell line was validated by respective manufacturers. ATCC validates their cell lines by human STR analysis. No additional authentication was performed.
Mycoplasma contamination	The cell line was routinely screened for Mycoplasma contamination (once monthly) and found to be negative.
Commonly misidentified lines (See ICLAC register)	None of the commonly misidentified cell lines were used in this study.

Animals and other organisms

Policy information about studies involving animals; ARRIVE guidelines recommended for reporting animal research

Laboratory animals	BALB/c mice, female, 8- to 10-week-old, were housed in conditions with controlled temperature (20-26°C), humidity (40–70%), and 12/12-hour dark/light cycle.
Wild animals	No wild animals were used in the study.
Field-collected samples	No field-collected samples were used in the study.
Ethics oversight	All mouse infection experiments were evaluated by the ethics committee of the Jinan University.

Note that full information on the approval of the study protocol must also be provided in the manuscript.

Flow Cytometry

Plots

Confirm that:

☒ The axis labels state the marker and fluorochrome used (e.g. CD4-FITC).

☒ The axis scales are clearly visible. Include numbers along axes only for bottom left plot of group (a 'group' is an analysis of identical markers).

☒ All plots are contour plots with outliers or pseudocolor plots.

☒ A numerical value for number of cells or percentage (with statistics) is provided.

Methodology

Sample preparation	2×106 exponentially grown C. albicans cells were stained with DCFDA (20 µg/ml) at 37°C for 20 min or JC-1 (20µM) at 37°C for 30 min in the dark, washed with PBS.
Instrument	BD FACSCanto
Software	BD FACSDiva v8.0.1
Cell population abundance	10000 cells were analyzed for each sample.
Gating strategy	We used FSC to exclude debris and cell fragments.

☒ Tick this box to confirm that a figure exemplifying the gating strategy is provided in the Supplementary Information.
